# Unveiling the Microbiota Diversity of the Xerophyte *Argania spinosa* L. Skeels Root System and Residuesphere

**DOI:** 10.1007/s00248-020-01543-4

**Published:** 2020-06-25

**Authors:** Francesca Mapelli, Valentina Riva, Lorenzo Vergani, Redouane Choukrallah, Sara Borin

**Affiliations:** 1 Department of Food Environmental and Nutritional Sciences, Università degli Studi di Milano, 20133 Milan, Italy; 2 Hassan II, Salinity and Plant Nutrition Laboratory, Institut Agronomique et Vétérinaire, Agadir, Morocco

**Keywords:** Argan, Microbiome, Plant growth–promoting bacteria, Litter, Soil, Sustainable agriculture

## Abstract

The microbiota associated to xerophyte is a “black box” that might include microbes involved in plant adaptation to the extreme conditions that characterize their habitat, like water shortage. In this work, we studied the bacterial communities inhabiting the root system of *Argania spinosa* L. Skeels*,* a tree of high economic value and ecological relevance in Northern Africa. Illumina 16S rRNA gene sequencing and cultivation techniques were applied to unravel the bacterial microbiota’s structure in environmental niches associated to argan plants (i.e., root endosphere, rhizosphere, root-surrounding soil), not associated to the plant (i.e., bulk soil), and indirectly influenced by the plant being partially composed by its leafy residue and the associated microbes (i.e., residuesphere). Illumina dataset indicated that the root system portions of *A. spinosa* hosted different bacterial communities according to their degree of association with the plant, enriching for taxa typical of the plant microbiome. Similar alpha- and beta-diversity trends were observed for the total microbiota and its cultivable fraction, which included 371 isolates. In particular, the residuesphere was the niche with the highest bacterial diversity. The Plant Growth Promotion (PGP) potential of 219 isolates was investigated in vitro, assessing several traits related to biofertilization and biocontrol, besides the production of exopolysaccharides. Most of the multivalent isolates showing the higher PGP score were identified in the residuesphere, suggesting it as a habitat that favor their proliferation. We hypothesized that these bacteria can contribute, in partnership with the argan root system, to the litter effect played by this tree in its native arid lands.

**Electronic supplementary material:**

The online version of this article (10.1007/s00248-020-01543-4) contains supplementary material, which is available to authorized users.

## Introduction

*Argania spinosa* L. Skeels is a xerophyte, endemic from Northern Africa and especially present in the Agadir area in South Western Morocco. Archaeobotanists demonstrated the high importance of this tree for the economy of Morocco since the past centuries [1]. Nowadays, the plant has a pivotal economic value for this country due to the production of argan oil that is worldwide requested by the cosmetic industry. Moreover, argan oil is traditionally used for food consumption, and it has been proposed as a nutraceutical since the characterization of the fruit flesh content led to identify several phenolic compounds with antioxidant activity [2, 3]. The leaves and fruits of argan tree are exploited as forage, and the plant is considered the base of a peculiar agrosystem, which includes argan tree, goat, and barley, having a great socioeconomic value for South Western Morocco [4].

Noteworthy, argan trees are able to grow on low fertile soils, mining ions, and nutrients from the deep soil layers and increase their concentration in the top soil as a consequence of litter deposition, i.e. litter effect [5]. Indeed, the use of litter composed by argan leaves as soil amendments is widespread among farmers in South Western Morocco according to tradition and the local availability of this organic fertilizer (Redouane Choukrallah, personal communication). The portion of soil that is more influenced by litter decomposition, or other organic supplement, is defined as “residuesphere” [6], and it is highly relevant in terms of soil fertilization because it represents the soil niche with highest mineralization rates [7]. Litter decomposition depends upon several factors including its origin (e.g., leafy substrate, woody debris, root materials), and a recent study showed that leaf litter plays the strongest plant growth promotion effect compared to the addition of root litter into the soil, likely due to the different carbon composition of these litter types [8]. Moreover, the types of climate can influence the litter decomposition, but information on this crucial process for soil fertility in dry climatic regions are scarce [9]. An aspect that has not yet been explored is the possible role played by the litter-associated microbes as plant growth promoters, in relation to the litter effect.

Plant growth and adaptation to the environmental conditions are strongly supported by the plant microbiome [10]. In the past years, extremophilic plants and their associated microbiota have been largely studied [11–13] aiming at the exploitation of beneficial microbe–plant interactions to boost plant growth and productivity under harsh conditions such as soil salinity [14–17] and water shortage [18–22]. In this framework, experimental protocols have been established in the last years to effectively combine plant seeds and extremophilic microbes that are able to cope with desiccation and to promote the plant growth under drought conditions [23]. Among extremophilic plants, xerophytes (e.g., cacti, argan, resurrection plants) are adapted to long-term survival under severe water scarcity, by means of several mechanisms like the decrease of transpiration surface and stomatal closure. Previous studies suggested a key role of endophytic bacteria in terms of plant adaptation to drought [24, 25]; however, studies on the diversity and composition of the microbiota associated to xerophytes are still scarce. Surprisingly, we realized that no data are available for the xerophilic species *A. spinosa* despite its economic value and the crucial ecological role it plays in the native region, where it represents a unique tool to counteract desertification [26].

In this work, we studied the microbiota inhabiting plant and soil fractions collected along a gradient that includes environmental niches (i) closely associated to *A. spinosa* plant (i.e., root endosphere, rhizosphere, root-surrounding soil), (ii) not associated to the plant (i.e., bulk soil), and (iii) indirectly influenced by the plant being partially composed by its leafy residue and the associated microbes (i.e., litter, from here on defined as residuesphere). The phylogenetic composition of the overall argan bacterial communities was disentangled by 16S rRNA gene high-throughput sequencing. Furthermore, we focused on the cultivable microbiota establishing a large bacteria collection which functional diversity was characterized in vitro in terms of Plant Growth Promotion (PGP) potential. These bacteria might be exploited in the future for research on plant adaptation under lack of water and the development of biofertilizers adapted to conditions of drought and soil salinity.

## Materials and Methods

### Root System Sampling and Processing

*Argania spinosa* L. Skeels root system was collected in a field located in the protected Argan forest within the farm of IAV Hassan II, the Horticulture campus of Agadir in the southern part of Morocco. The permission to conduct the sampling was granted by Professor Redouane Choukr-Allah. The local climate is arid Mediterranean, with an average annual rainfall of 200 mm restricted to the winter months (December–January). The texture of the soil in the sampling site is loamy sand, moderately rich in organic matter, and has pH 8.5 with a saturated past conductivity of 1.8 [27]. Plant cover in the sampling site was patchy and limited to *A. spinosa* plants, considering an average 10-m distance between groups of plants. Argan roots with soil particles adhering to the root surface were collected at 30-cm depth (E + R fractions). Roots were shaken and the soil not tightly attached to the root system was gathered and defined as root-surrounding soil (SSR fraction, [28]). Bulk soil, i.e., the portion of soil not influenced by any plant root exudates, was collected at 5-cm depth a distance of 2 m from each argan tree, where no visible plants or stones were present (B fraction). Residuesphere, i.e., the portion of soil influenced by the decomposition of residue ([6]; in our study it was composed by a mixture of leaf litter and soil), was collected below the tree crown at 3–5-cm depth after the removal of the surface material (Re fraction). Replicate samples were collected from three different argan trees (*n* = 3 per each fraction in total) using sterile tools and were processed within 24 h from the time of collection. Rhizosphere soil was separated from the sampled roots in sterile conditions, and the clean roots were then surface sterilized, as previously described [29]. Five washes with sterile water were performed to remove any trace of the reagents used. The wash solution from the last rinse was cultured in plates containing 1:10 869 medium [30, 31] to determine the efficacy of sterilization. Before DNA extraction and bacteria cultivation, soil and residuesphere fractions were carefully stirred and homogenized using a sterilized spatula. After the preparation described above, all samples were immediately used for bacteria cultivation and stored at − 20 °C until DNA extraction and molecular analyses were applied.

### Metagenomic DNA Extraction

For the rhizosphere (*n* = 3), root-surrounding soil (*n* = 3), bulk soil (*n* = 3), and residuesphere (*n* = 3) fractions, the metagenomic DNA was extracted from 0.5 g of sample using the PowerSoil DNA Isolation Kit (MoBio Inc., CA, USA). To obtain metagenomic DNA of endophytes, 1 g of the root (*n* = 3) was surface-sterilized (as described above) and crushed using liquid nitrogen as previously reported by Cherif et al. [29]. The DNA was extracted using a DNeasy Plant Max Kit (Qiagen). The DNA concentration of each sample was assessed using a Qubit™ flurometer with dsDNA HS kit (ThermoFisher).

### Quantification of Bacteria by Quantitative PCR (qPCR)

qPCR reactions were performed on metagenomic DNA in polypropylene 96-well plates using a BIORAD CFX Connect™ Real-Time PCR Detection System by the amplification of 16SrRNA universal bacterial gene using primers 357F (5’-CCCTACGGGAGGCAGCAG-3′) and 907R (5’-CCGTCAATTCCTTTGAGTTT-3′) [32] with the following conditions: 0.3 μM of each primer, 7.5 μl SsoAdvanced™Universal SYBR®Green Supermix (BIORAD), 1 μl DNA template, 15 μl final volume. PCR thermal conditions were 3 min at 98 °C, followed by 35 cycles of 98 °C for 1 min, 30 s at 58 °C, and 72 °C for 1 min. Standards were prepared through ten-fold serial dilutions of the plasmid pCR®II-TOPO® carrying the 16S rRNA gene of the strain *Asaia stephensi* [33] and cloned into TOP10 *Escherichia coli* competent cells (TOPO® TA Cloning® Kit, ThermoFischer Scientific). The plasmids were isolated from LB overnight cultures of the transformant *E. coli* and quantified using the Qubit dsDNA HS Assay Kit (Thermo Fisher Scientific) to determine the number of 16S rRNA copies contained. Standard curves were constructed with a series of dilutions ranging from 2 × 10^8^ to 2 × 10^4^ 16S rRNA copies per microliter. All the standards and the samples were run in triplicate. R^2^ and amplification efficiency of the qPCR assay were 1000 and 90% respectively. Statistical analysis of qPCR results was performed by a one-way ANOVA test using the aov function of the R software (R version 3.6.1, base package, [34]).

### Illumina High-Throughput Analysis of 16S rRNA Gene

Illumina tag analysis (MiSeq 300 × 2 Paired End) of the V3-V4 hypervariable regions of the 16S rRNA gene was performed on the metagenomic DNA by BioFab (Italy), using primers IlluminaF (TCGTCGGCAGCGTCAGATGTGTATAAGAGACAGCCTACGGGNGGCWGCAG) and IlluminaR (GTCTCGTGGGCTCGGAGATGTGTATAAGAGACAGGACTACHVGGGTATCTAATCC) (http://support.illumina.com/downloads/16s_metagenomic_sequencing_library_preparation.html; [35]). The obtained sequences were analyzed using a combination of the VSEARCH [36] and the QIIME v1.9 [37] software. Raw forward and reverse reads for each sample were assembled into paired-end reads considering a minimum overlapping of 50 nucleotides and a maximum of one mismatch within the region using PEAR—Paired-End reAd mergeR (https://sco.h-its.org/exelixis/web/software/pear/doc.html). The paired reads were then quality filtered discarding reads with a Phred quality score ≤ Q30, the primer sequences were removed and the individual sample files were merged in a single fasta file. Chimeras were removed using both de-novo and reference-based detection. For reference chimera detection, the “Gold” database containing the chimera-checked reference database in the Broad Microbiome Utilities (http://microbiomeutil.sourceforge.net/) was used. After quality check and chimera removal, a total of 393.344 high-quality merged paired-end reads with an average length of 425 bp were obtained. Rarefactions were assessed: all samples had a coverage of more than 99% (Supplementary Figure 1). QIIME was used to generate the operational taxonomic units of 97% sequence identity (OTU_97_). Taxonomy was assigned to the representative sequences of the OTUs in QIIME using UClust [38] and searching against the latest version of the SILVA database 128 [39]. Finally, an OTU table (i.e., a sample x OTU count matrix with a tab containing the taxonomic affiliation of each OTU) was created. The OTU table and the phylogenetic tree were calculated with FastTree2 [40] using default parameters and the PyNast-aligned representative sequences as an input. The OTU table and the phylogenetic tree were used as inputs for the subsequent analyses of alpha- and beta-diversity. The sequence reads were deposited in the NCBI SRA database under the BioProject ID: PRJNA484110.

### Bacteria Isolation, Cultivation, and Identification

Bacteria were isolated from each plant/soil/residuesphere fraction, after pooling, and homogenization of the samples collected from the replicate plants (*n* = 3). For endophytic bacteria isolation, the root tissues were smashed with sterile mortar and pestle after the above-described sterilization procedure. One gram of the resulting soil/root tissue was suspended in 9 ml of physiological solution (0.9% NaCl), diluted in 10-fold series and plated on 1:10 869 medium [30] supplemented with cycloheximide 0.1 g/L to prevent fungal growth. Each decimal dilution was plated in triplicate to count the colony-forming unit (cfu) and calculate the cultivable bacteria abundance (cfu/g) present in each fraction. Medium 869 has been proposed among the optimal media for the isolation of endophytes [30, 31], and it was also successfully applied to isolate rhizosphere bacteria [41]. Colonies were randomly picked after two days of incubation at 30 °C and were spread three times on the same medium to obtain pure cultures. The purified strains were stored at − 80 °C in 1:10,869 medium supplemented with 25% glycerol for later use. A collection of 371 bacterial isolates was obtained. Strain code includes information on the plant species (“A” for *Argania spinosa*), the medium used for the isolation (“8” for 1:10,869) and the fraction (“E/R/SSR/B/Re” for root endosphere/rhizosphere/root surrounding soil/bulk soil and residuesphere) followed by progressive numbers. The genomic DNA of each isolate was extracted through boiling cell lysis and the bacteria collection has been de-replicated by ITS-PCR fingerprinting (16S–23S rRNA Internal Transcribed Spacer-PCR, [42]) using ITS-F (5’-GTCGTAACAAGGTAGCCGTA-3′) and ITS-R (5′-GCCAAGGCATCCACC-3′) primers as previously described [43]. Bacterial isolates were grouped according to their ITS-PCR fingerprint profile, and one representative strain per “ITS group” has been selected for subsequent physiological characterization and phylogenetic identification. Bacterial strains were identified through 16S rRNA gene amplification and partial sequencing (Macrogen, Republic of South Korea) as previously described [43]. The 16S rRNA gene sequences of the bacterial isolates were subjected to BLAST search (using blastn program) and were deposited in the European Nucleotide Archive under the accession numbers LS991221–LS991231 (root endosphere), LS991172–LS991220 (rhizosphere), LS991066–LS991120 (root surrounding soil), LS991015–LS991065 (bulk soil), and LS991121–LS991171 (residuesphere).

### In Vitro Characterization of the PGP Potential of Cultivable Bacteria

In vitro screening for the presence of activities related to plant growth promotion (PGP) was performed for one representative strain per “ITS group” identified in the bacteria collection (*n* = 219). The solubilization of inorganic phosphate and the production of siderophores, ammonia, protease and exopolysaccharides (EPS) were assessed as described in detail by Cherif et al. [29]. The production of esterase was conducted using tributyrin agar plates, and scoring the strains as positive in the presence of a solubilization halo, as previously described [44]. For each isolate, we calculated a “PGP score” indicating the total number of positive activities according to the results of the PGP assays. The number of isolates positive to each performed PGP in vitro tests were accounted for each taxonomic group (i.e., Family) present in the bacteria collection and visualized as a heat map [45].

### Diversity and Phylogenetic Composition of the Microbiota and Statistical Analyses

The phylogenetic compositional differences of the bacterial communities inhabiting the different types of samples analyzed in this study were investigated both on the cultivable fraction (based on the 16S rRNA sequences of the isolated strains, *n* = 371) and on the entire bacterial microbiota (based on the Illumina 16S rRNA gene dataset). For the cultivable bacteria, the 16S rRNA gene sequences of each “ITS group” representative strain were aligned using the Clustal X software [46], and the output file was used to define operational taxonomic units at 97% of identity (OTU_97_) using DOTUR [47]. On the other hand, to test the differences of the overall bacterial composition among the fractions, we performed a Permutational multivariate analysis of the variance (PERMANOVA) on the Bray–Curtis distance matrix generated from the Illumina 16S rRNA gene dataset, considering the “Fraction” (five levels: “Endosphere”, “Rhizosphere”, “Soil Surrounding Root”, “Bulk soil”, and “Residuesphere”) as categorical variable. Bray–Curtis distance matrix was used also to perform a Principal Coordinates Analysis (PCoA) and a Canonical Analysis of Principal coordinates (CAP). Statistical analyses were conducted in PRIMER v. 6.1, PERMANOVA++ for PRIMER routines [48]. Richness, i.e., number of OTU_97_, Shannon, and dominance indices were calculated using the PAST software [49] and their statistical difference was evaluated using the R software version 3.6.1 [34]. After normality and homoscedasticity assessment, we applied Tukey’s Ladder of Powers transformation using the “transformTukey” function within the package rcompanion [50]. Normalized data were then subjected to the analysis of variance (ANOVA) and Tukey–Kramer mean grouping considering the index as response variable and “Fraction” as explanatory categorical variable. The α-diversity index values generated by the Illumina dataset and the detailed results of the ANOVA analysis are available within the Dataverse “madforwater-wp3” created by the University of Milan at the following link: 10.13130/RD_UNIMI/JAG2BM.

## Results and Discussion

### Phylogenetic Composition and Diversity of Bacterial Communities Associated to *Argania spinosa* L. Skeels

**Fig. 1:**
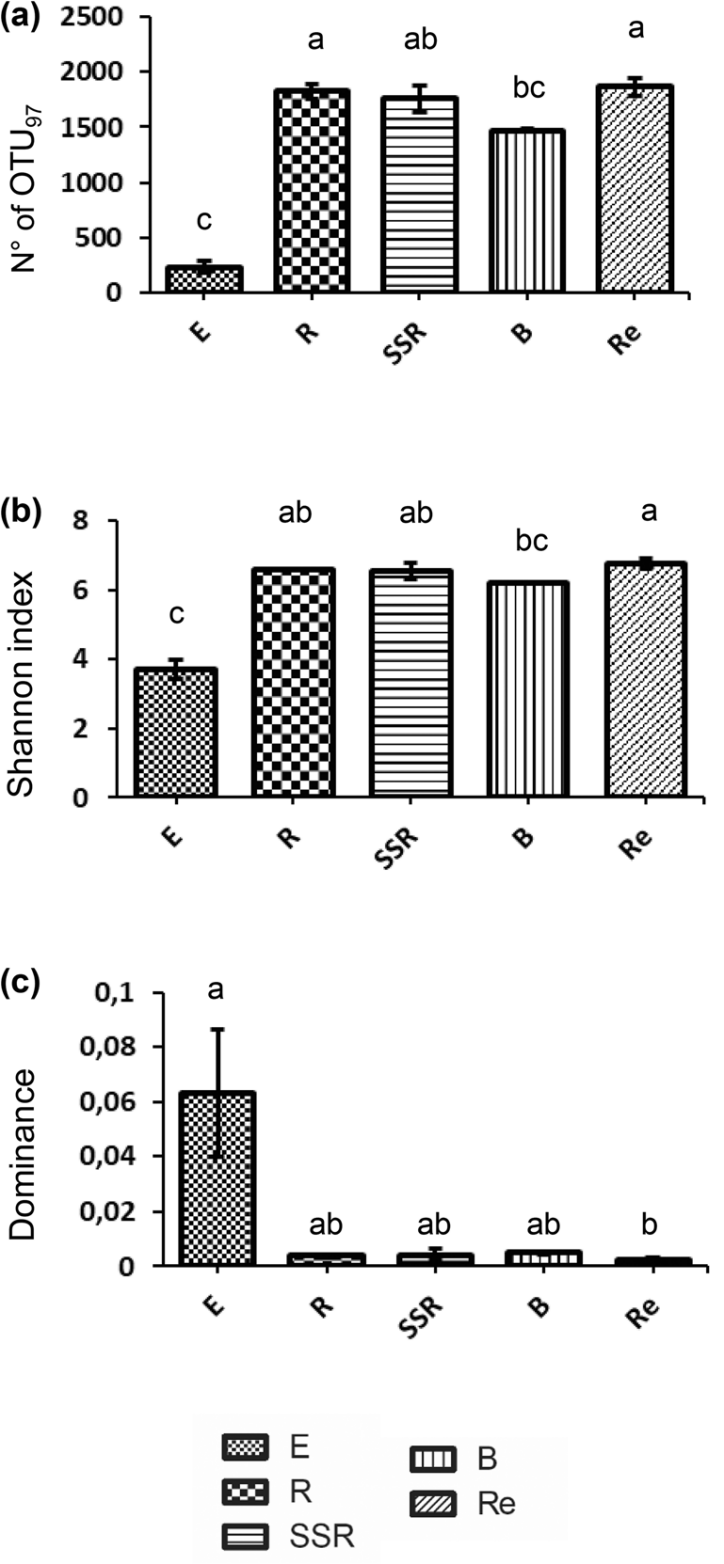
α-Diversity indices of the total bacterial communities associated to *Argania spinosa* (**a**) Richness, expressed as number of OTU_97_, (**b**) Shannon index, and (**c**) Dominance. The indices were calculated from the OTU_97_ table generated by 16S rRNA gene Illumina sequencing of the bacterial communities inhabiting the root endosphere (E), rhizosphere (R), root-surrounding soil (SSR), bulk soil (B), and residuesphere (Re) fractions. Letters indicate the statistical differences among the fractions, according to the analysis of variance (ANOVA)

The structure of the overall bacterial communities associated to the *A. spinosa* root system (E; R, SSR fractions), bulk soil (B), and residuesphere (Re) was disentangled by Illumina sequencing of 16S rRNA gene. For the endosphere fraction, the 16S rRNA Illumina sequencing was successful only for two of the three analyzed replicates. A total of 2236 OTU_97_ were identified, the rarefaction curves of the libraries were assessed, and all samples had a coverage of more than 99% (Supplementary Figure 1). The number of OTU_97_ (richness) was significantly lower (*p* < 0.05) in the endophytic bacterial community (236 ± 51) compared to the other fractions except bulk soil (R: 1825 ± 66, SSR: 1759 ± 119, B: 1468 ± 12, Re: 1864 ± 80) as shown in Fig. 1a. Likewise, the endophytic bacterial community was characterized by significantly lower diversity (according to the Shannon index values, *p* < 0.01 except in comparison to bulk soil, Fig. 1b) and a higher dominance (Fig. 1c) of few bacterial populations, although the latter result is corroborated by statistical significance (*p* < 0.05) only in comparison to residuesphere fraction. The lower richness and diversity of the root endosphere bacterial community is in agreement with previous reports on the model plant *A. thaliana* [51] and different crops growing under both conventional agriculture and desert farming conditions [28, 52, 53]. Bulk soil, i.e., the portion of soil not influenced by root exudates, hosted a significantly less rich (*p* < 0.05) bacterial community compared to the rhizosphere (R) and residuesphere (Re) fractions (Fig. 1a) while the highest diversity was detected in the bacterial community that inhabits the residuesphere (Fig. 1b). The lower richness of the bacterial community inhabiting the bulk soil compared to those colonizing the rhizosphere is common in desertic areas [54–57], in contrast to what is generally reported for conventional agricultural systems [58]. Indeed, it has been proposed that the nurturing effect played by plants on soil bacterial communities becomes more evident under harsh conditions, favoring the establishment of a more diverse and rich assembly of bacterial populations around plant roots due to higher nutrient availability (i.e., root exudates) compared to the nutrient-poor desertic soil [57].

**Fig. 2:**
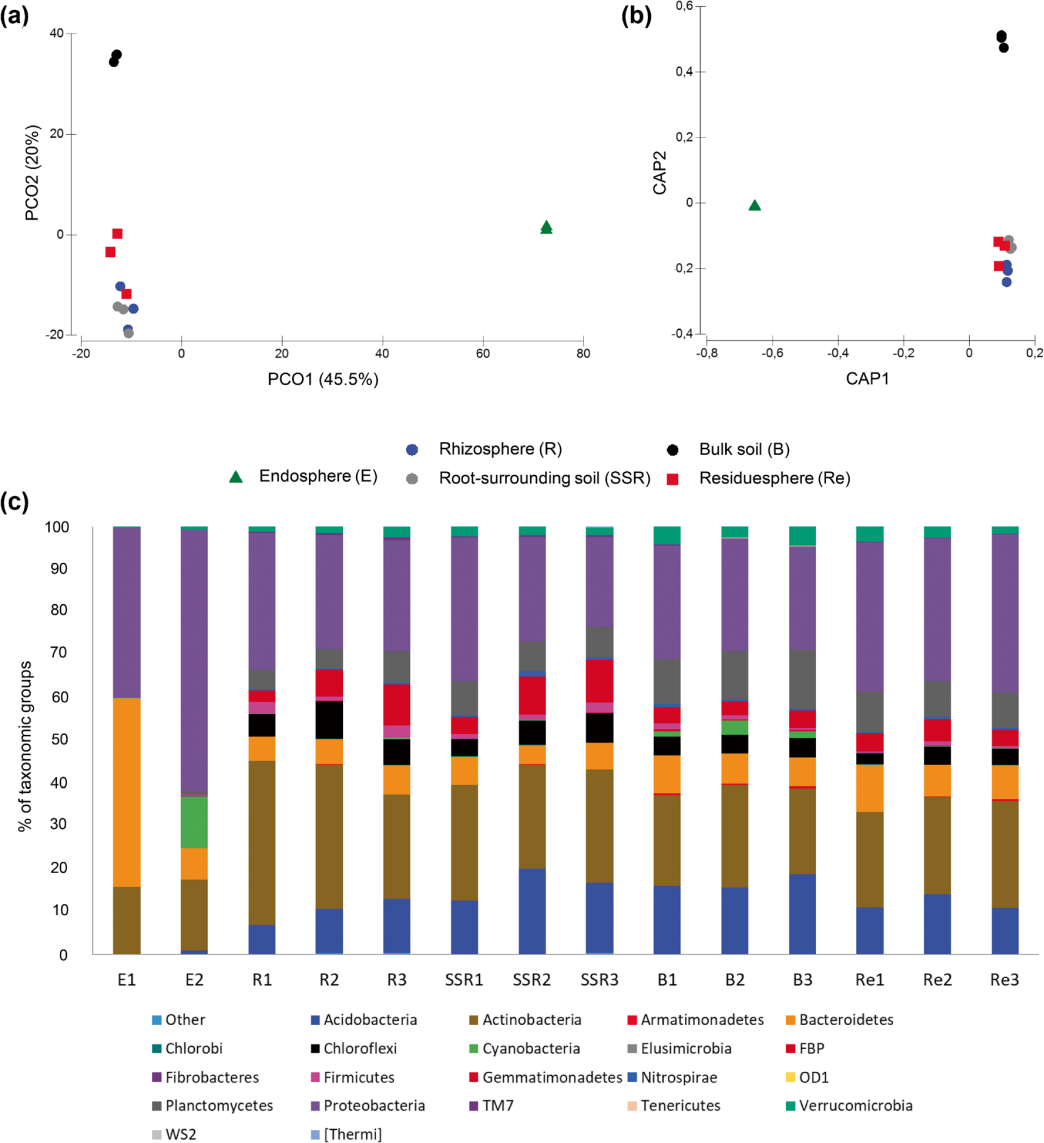
Phylogenetic classification and β-diversity of the total bacterial communities associated to *Argania spinosa*. **a** Principal coordinates analysis (PCoA) and (**b**) constrained analysis of principal coordinates (CAP) of the bacterial communities inhabiting the *A. spinosa* root endosphere (E), rhizosphere (R), root-surrounding soil (SSR), bulk soil (B), and residuesphere (Re) fractions. PCoA and CAP were calculated from the OTU_97_ table generated by 16S rRNA gene Illumina sequencing. **c** Relative abundance of different taxonomic groups (at phylum level) in the bacterial communities of each sample of the fractions E, R, SSR, B and Re

The Principal Coordinate Analysis (PCoA) and the Constrained analysis of principal coordinates (CAP) showed that the bacterial communities are clustered according to the fraction type, and unveiled a clear separation of the root endosphere (E) and bulk soil (B) bacterial communities compared to those inhabiting the R, SSR, and Re niches (Fig. 2a,b). The categorical variable “Fraction” significantly influenced the composition of the bacterial communities in the analyzed samples (PERMANOVA, F_4, 9_ = 6.27; *p* = 0.001). In particular, as it could be inferred by the PCoA, the pairwise PERMANOVA results confirmed that the root endosphere and the bulk soil hosted significantly different assembly of bacterial populations while the bacterial microbiota of the rhizosphere, root-surrounding soil and residuesphere could not be significantly distinguished (Table 1). The beta-diversity observed reflects the selective effect played by the plant through the release of root exudates and the complex signaling mechanism it establishes with the soil-dwelling microflora. In fact, plants are known to actively select rhizosphere-competent bacterial populations (R and SSR fractions) starting from the initial pool of bacteria present in the bulk soil (B), employing an array of molecules (e.g., flavonoids, volatile organic compounds) to attract beneficial microorganisms able to promote plant growth and control pathogens in proximity of its root system and establish mutualistic interactions with them [59–61]. Moreover, among the rhizosphere and rhizoplane colonizers, only a subset of bacterial populations is able to enter the root tissues showing an endophytic lifestyle [62], and the sharp separation of the endosphere bacterial communities from those inhabiting the soil fractions and the residuesphere (Fig. 2a,b; *p* < 0.05; Table 1) highlights a high specialization of the dominant taxa in root tissues of *A. spinosa*. Bacteria able to pass through the rhizoplane, which has been referred as a gate controlling the entry in the root [52], are endowed with specific traits that allow them to adapt to the endosphere environmental conditions and their assembly is finely regulated by the plant itself, as a response to specific needs such as pathogen protection [63]. In our study, the bacterial communities inhabiting the residuesphere samples could not be significantly discriminated from those associated to the plant roots, i.e., the rhizosphere and root-surrounding soil. The residuesphere analyzed in this study was a mixture of argan leafy substrate and soil, collected below the tree crown, and according to the results of the PCoA, we hypothesize a selective effect played by the plant on the residuesphere-colonizing bacterial community (Fig. 2a,b). Although less studied compared to the plant microbiome, it is known that litter types influence the structure of microbial communities [8] and a recent study showed that also the twig diameter shapes the fungal and bacterial assemblages associated to litter [64].

**Table 1:** Pairwise PERMANOVA for the 16S rRNA gene-based Illumina dataset

Groups	T	*P*
E, R	2.6707	0.019*
E, SSR	2.6055	0.026*
E, B	3.5484	0.009*
E, Re	3.0397	0.018*
R, SSR	0.92395	0.492
R, B	2.7382	0.009*
R, Re	1.4644	0.119
SSR, B	2.6329	0.011*
SSR, Re	1.4192	0.156
B, Re	3.0824	0.01*

Post-hoc test performed on the distance matrix generated according to OTU_97_ distribution of bacterial communities (16S rRNA gene-based Illumina dataset) in the different fractions (“E”, “R”, “SSR”, “B”, “Re”).

*E* endosphere, *R* rhizosphere, *SSR* root-surrounding soil, *B* bulk soil, *Re* residuesphere

* Indicates significant differences between samples

In the argan root system, bacterial phylogenetic groups were differently distributed, as particularly evident in the case of the root endosphere microbiota that was dominated by Proteobacteria, Bacteroidetes, and Actinobacteria (Fig. 2c), in accordance with previous high-throughput taxonomic characterization of the endophytic bacterial communities [51, 52, 65]. The prevalence of Proteobacteria and Actinobacteria was observed also in the soil and residuesphere fractions, while the Bacteroidetes phylum abundance decreased in comparison with the root endosphere. Interestingly, according to the results of a study that compare the bacterial communities of some crop plants and their wild relative, Bacteroidetes is one of the plant microbiome phyla which abundance in the root or rhizosphere seems to be more influenced by domestication [66], although its possible effect on plant performance is still unresolved [63]. Other phyla, such as Chloroflexi, Gemmatimonadetes, Verrucomicrobia, and Acidobacteria, which were not detected or were present below 1% of the total community in the root endosphere, were important components of the bacterial communities in the different soil fractions (R, SSR, B) and the residuesphere (Re) as shown in Fig. 2c. These Phyla are known to colonize soil much more efficiently than root surface and interior tissues [51, 52]. For example, Chloroflexi and Acidobacteria were among the most abundant taxa in soils associated to cork oak, a tree able to tolerate drought, sampled in different climatic regions [67]. Furthermore, the relative abundance of Chloroflexi and Acidobacteria over the total bacterial community was significantly higher in rhizospheric soil compared to the root endosphere in a study that extensively analyzed the microbiome of grapevine plants [53]. Chloroflexi, Verrucomicrobia, and Acidobacteria are oligotrophic Phyla [63] and their relative abundance increases across soil formation gradients, in agreement with the ability of certain phyla members to degrade recalcitrant carbon compounds that are more abundant in mature soils [57, 68]. The different Classes of the Proteobacteria phylum were unevenly distributed in the root endosphere and the plant associated soils (R, SSR), bulk soil, and residuesphere (Supplementary Table 1). Gammaproteobacteria and Betaproteobacteria were mostly retrieved from the endosphere metagenome and decreased in the other fractions (Supplementary Table 1). Gammaproteobacteria was reported as the dominant taxon within the endosphere described in both leaves and branches for different arborous crops such as the *Olea* and *Citrus* genera [69]. In grapevine root endosphere, Gammaproteobacteria were among the dominant classes and, according to the predicted functional potential of the bacterial populations, considered possibly related to several plant growth promoting traits as well known for several of their cultivable representatives [53]. On the opposite, Alphaproteobacteria dominated the Proteobacteria phylum in the soil and residuesphere fractions (Supplementary Table 1). The phylogenetic affiliation of OTU_97_ in the residuesphere, with a prevalence of Proteobacteria and Actinobacteria, resembles that recently reported for the litter of different tree species [70]. Proteobacteria classes in the residuesphere niche can change in relative abundance during the decomposition process, providing indirect indications on the chemical composition shift of the substrate [64]. In particular, a higher abundance of Alphaproteobacteria, the same class retrieved in the argan Re samples, was reported in the later phases of *Salix caprea* litter decomposition [64] when recalcitrant molecules become more abundant and favor the enrichment of slow-growing taxa belonging to the class Alphaproteobacteria.

**Fig. 3:**
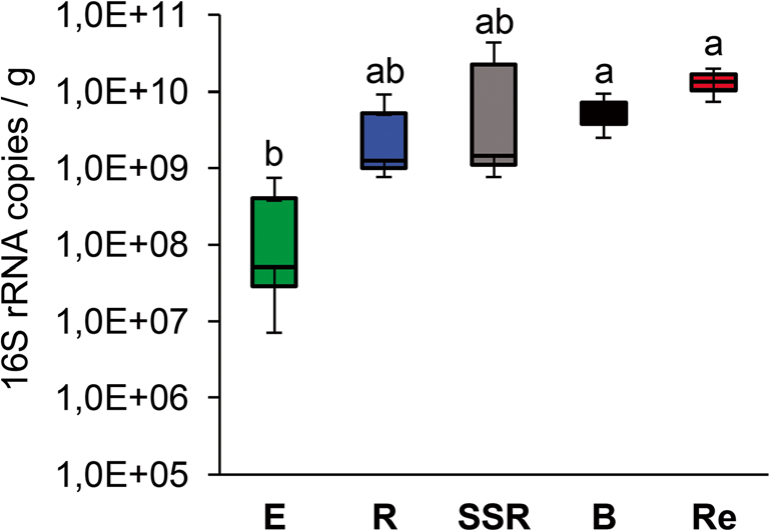
Evaluation of bacterial population abundance in the different plant, soil, and residuesphere fractions through qPCR. The graph indicates the number of amplified 16S rRNA gene copies per gram of root tissue in the endosphere (E) or per gram of soil in the rhizosphere (R), root-surrounding soil (SSR), bulk soil (B) and residuesphere (Re) fractions. Letters indicate the statistical differences in 16S rRNA gene abundance among the fractions, according to the analysis of variance (ANOVA)

Besides the significant differences observed comparing alpha- and beta-diversity values and the phylogenetic structure of the bacterial communities colonizing the different plant, soil, and residuesphere niches, the concentration of the 16S rRNA gene amplified from the extracted metagenome (Fig. 3) was significantly different according to the “Fraction” factor (ANOVA, *p* = 0.020, Table 2). However, the pairwise test performed on these data revealed that the bacterial abundance was significantly different only comparing the root endosphere fraction to the bulk soil (*p* = 0.046) and residuesphere (*p* = 0.015), without differences in the estimated bacterial abundance between rhizosphere, root-surrounding soil, and bulk soil (Table 3). This result contrasts the general knowledge on the rhizosphere enrichment effect on the microbial community that leads to a higher density of bacteria in plant-associated soil fractions fueled by carbon substrates released by roots [71]. Our relatively small set of data did not allow us to elaborate a convincing explanation to justify the observed deviation from an effect that has been described independently from the plant species and also in ecosystems subjected to different stress types like pollution [32] and xeric conditions [72]. Given the scarcity of data on bacterial abundance associated to the root system of xerophytes, we can hypothesize that tree species such as *A. spinosa* present a rhizodeposition profile able to select a peculiar microbiota from the bulk soil but not sufficient to sustain the quantitative enrichment conventionally observed in the rhizosphere niche. Additional studies on drought tolerant trees growing in different soil types could provide further evidences to support this hypothesis.

**Table 2:** Main test comparison of the bacterial population abundance in the different plant and soil fractions evaluated as 16S rRNA copies number by qPCR according to fraction types

	Df	Sum_Sq	Mean_Sq	F_value	Pr(>F)
Fraction	4	92.354	230.885	48.466	0.01962*
Residuals	10	47.639	0.47639		

*E* endosphere, *R* rhizosphere, *SSR* root-surrounding soil, *B* bulk soil, Re residuesphere.

* Indicates significant differences between samples

**Table 3:** Pairwise comparison of the bacterial population abundance in the different plant and soil fractions evaluated as 16S rRNA copies number by qPCR according to the fraction types

Fraction	diff	lwr	upr	*p*
E-B	− 18,831,730	− 37,378,665	− 0.02847953	0.0462268*
R-B	− 0.3737773	− 22,284,708	148,091,617	0.9600296
Re-B	0.4039798	− 14,507,137	225,867,334	0.9478002
SSR-B	− 0.1323617	− 19,870,552	172,233,184	0.9992018
R-E	15,093,957	− 0.3452978	336,408,920	0.1278523
Re-E	22,871,529	0.4324594	414,184,637	0.0152834*
SSR-E	17,508,114	− 0.1038821	360,550,487	0.0665269
Re-R	0.7777572	− 10,769,363	263,245,067	0.6523767
SSR-R	0.2414157	− 16,132,778	209,610,917	0.9918603
SSR-Re	− 0.5363415	− 23,910,350	131,835,199	0.8701704

*E* endosphere, *R* rhizosphere, *SSR* root-surrounding soil, *B* bulk soil, Re residuesphere.

* Indicates significant differences between samples

### Phylogenetic Classification and Distribution of the Cultivable Bacteria Associated to *A. spinosa* Root System, Bulk Soil, and Residuesphere

**Fig. 4:**
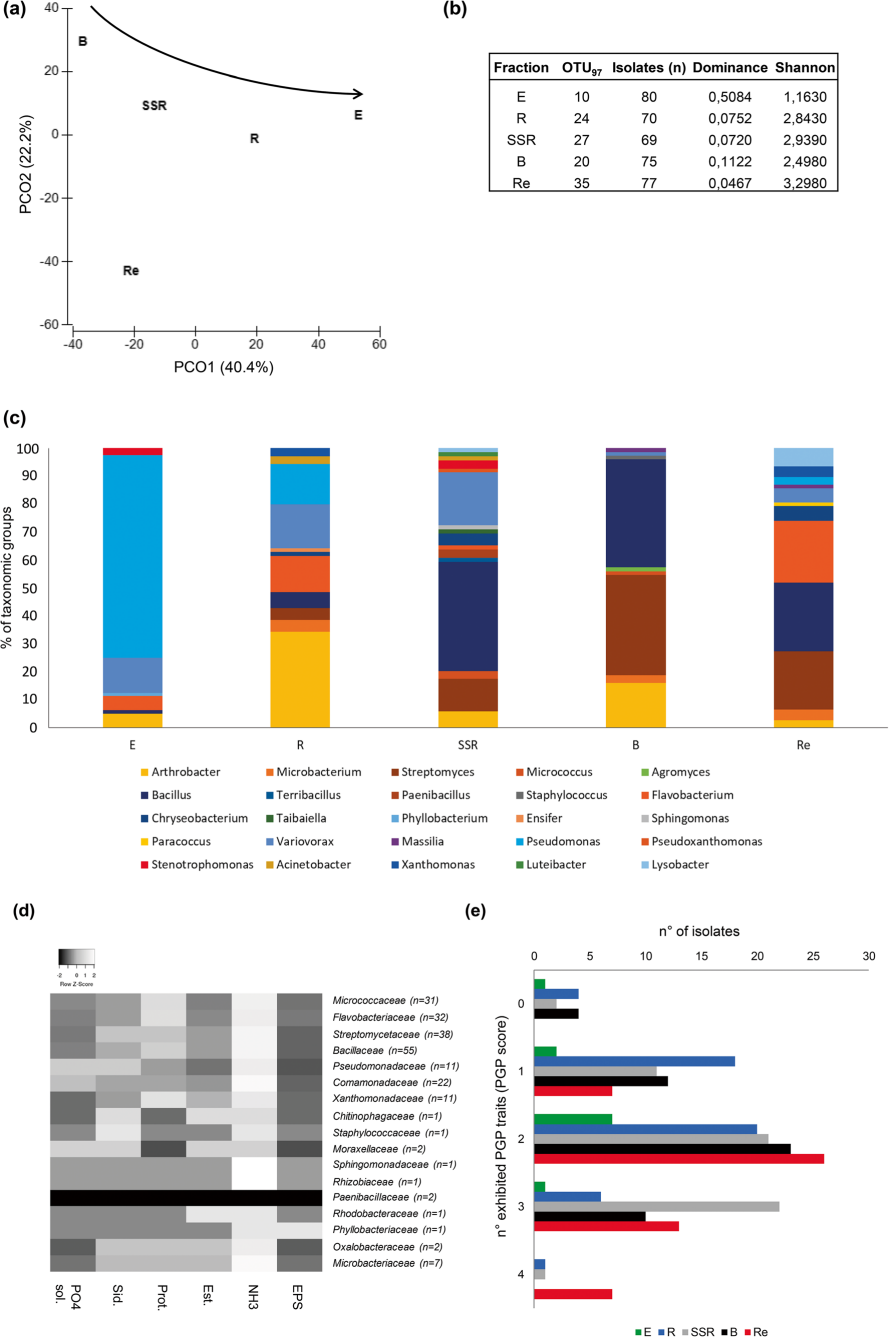
Cultivable bacteria isolated from *Argania spinosa* root system, bulk soil, and residuesphere. **a** Principal coordinates analysis (PCoA) of the cultivable bacterial communities inhabiting the *A. spinosa* root endosphere (E), rhizosphere (R), root-surrounding soil (SSR), bulk soil (B), and residuesphere (Re) fractions. PCoA was calculated from the OTU_97_ table obtained from the 16S rRNA gene sequences of the isolates. The analysis has been performed on the entire bacteria collection (*n* = 371). The arrow indicates the increasing level of relationship between the plant and the bacterial assemblages. **b** α-Diversity indices calculated on the cultivable bacterial communities. **c** Relative abundance of bacterial genera in the cultivable communities isolated from the different fractions. **d** Heat map showing the distribution of the PGP activities in the different bacterial families present in the collection (the number of tested isolates per each family is indicated in brackets). **e** Number of plant growth promotion traits (“PGP score” ranging from 0 to 4) exhibited by the cultivable bacteria of the collection. Results accounted the number of isolates positive to the different performed PGP tests and are represented according to the isolation fractions: root endosphere (E), rhizosphere (R), root-surrounding soil (SSR), bulk soil (B), and residuesphere (Re)

There are many evidences that plant-associated bacteria are able to sustain plant growth under adverse environmental conditions [13, 73]; however, no report exists in the literature on the identity and the PGP potential of the argan tree–associated bacteria. We established a collection of 371 bacterial isolates from the root system (E-R-SSR fractions) of *A. spinosa*, its residuesphere and the bulk soil, using the same cultivation medium for all the fractions in order to compare their composition in terms of cultivable bacteria taxonomy. With this aim, we selected a medium previously applied for the isolation of both endophytes and rhizospheric bacteria [30, 31, 41]. We avoided media mimicking the specific conditions of the different niches (e.g., endosphere, rhizosphere, soil) which, by the application of selecting cultivation conditions (e.g., the use of saline medium) could hamper the comparison between the niche sub-collections. The sequencing of the 16S rRNA gene of the isolates allowed to (i) identify phylogenetically the bacteria (Supplementary Table 2) and (ii) cluster the cultivable bacteria in OTU_97_ (according to the % of identity of their 16S rRNA gene sequence). A PCoA was performed on the cultivable microbiota OTU_97_ to visualize the differences between the analyzed fraction. PCoA showed that the samples are distributed according to the level of association to the *A. spinosa* root system, with the bacteria isolated from the residuesphere that are more diverse from those isolated from the other niches, including both the soil fractions and endosphere (Fig. 4a). A similar trend was observed by investigating the cultivable bacterial community of plants grown under drought conditions [28], although residuesphere was not included in the analysis. The 371 bacterial isolates were uniformly distributed among the five sub-collection (i.e., number of isolates per fraction, Fig. 4b) established from the different microhabitats of origin, where a similar titer (ranging between 1.13 × 10^7^ and 7.08 × 10^7^ cfu/g, Figure S2) of bacteria cultivable under the applied conditions was retrieved. We observed for the cultivable microbiota a trend of the alpha-diversity indices similar to that calculated on the 16S rRNA Illumina dataset. For example, the richness (number of OTU_97_) and the Shannon indices were lower in the endophytic bacteria sub-collection compared to the soil and residuesphere ones (Fig. 4b), in agreement with what detected for the overall bacterial community composition. Similarly, the residuesphere sub-collection had a higher number of OTU_97_ and Shannon index (Fig. 4b), in coherence with the data obtained on the 16S rRNA Illumina dataset in particular for the latter ecological index. Discrepancies can be observed between the composition of total bacterial communities and their cultivable fraction. This is nevertheless expected, considering the so-called “great plate count anomaly” [74] and the selection effect imposed by the application of one cultivation medium.

The presence and the distribution of the bacterial genera in the collection established from the root (E), soil (R, SSR, B) and residuesphere (Re) fractions reflected the same pattern obtained by the alpha-diversity indices calculated by the OTU_97_ clustering. The phylogenetic composition of the cultivable communities showed that R, SSR, and B soil fractions were overall more similar compared to the E and Re sub-collections and that the root endosphere hosted a clearly different composition of cultivable bacteria compared to the other environmental niches (Table 4, Fig. 4c). Moreover, the residuesphere and the root-surrounding soil hosted the richest and more diverse bacterial community in terms of bacterial genera (Fig. 4c). The richness of OTU_97_ detected both in the total and cultivable fraction of the bacterial microbiota associated to the argan residuesphere, besides the high taxonomic diversity of the corresponding sub-collection, suggests that this niche could potentially harbor novel microbial resources of interest for a tailored management of arid lands in the frame of sustainable agriculture. As shown in Fig. 4c, and in Table 4, the bacteria isolated from the root endosphere mainly belonged to the genus *Pseudomonas*, making the Class Gammaproteobacteria the most abundant within the cultivable endophytes of *A. spinosa*, in accordance with the taxonomic classification of endophytes selected by other plants, i.e., date palms, in desert-farming agro-ecosystems [29]. Moreover, this is in agreement with the high percentage of Gammaproteobacteria OTU_97_ identified through 16S rRNA Illumina sequencing in the *A. spinosa* microbiota. On the contrary, though the Alphaproteobacteria class was abundantly retrieved in the 16S rRNA Illumina libraries generated from soil fraction and residuesphere, its representatives were scarcely recorded in the bacteria collection. Indeed, only 4 isolates were classified as Alphaproteobacteria, and they were divided in 4 genera (i.e., *Phyllobacterium*, *Ensifer*, *Sphingomonas*, *Paracoccus*) each peculiar of a different niche (Table 4). Likewise, the Bacteroidetes phylum was scarcely represented (*n* = 4) in the endosphere sub-collection of isolates while it became more relevant in the residuesphere collection that included 17 isolates of the *Flavobacterium* genus (Table 4). Actinobacteria were mainly isolated from the *A. spinosa* rhizosphere (*n* = 30) and bulk soil (*n* = 43), where the most abundant genera were *Arthrobacter* and *Streptomyces*, respectively (Table 4).

**Table 4:** Phylogenetic affiliation of the cultivable bacteria associated to the *Argania spinosa* root system (E, R, SSR fractions), bulk soil (B), and residuesphere (Re)

Phylum/Class	E	R	SSR	B	Re	Genus	E	R	SSR	B	Re
Actinobacteria	4	30	14	43	21	*Arthrobacter*	4	24	4	12	2
						*Microbacterium*	0	3	0	2	3
						*Streptomyces*	0	3	8	27	16
						*Micrococcus*	0	0	2	1	0
						*Agromyces*	0	0	0	1	0
Bacilli	1	4	30	30	19	*Bacillus*	1	4	27	29	19
						*Terribacillus*	0	0	1	0	0
						*Paenibacillus*	0	0	2	0	0
						*Staphylococcus*	0	0	0	1	0
Bacteroidetes	4	10	5	0	21	*Flavobacterium*	4	9	1	0	17
						*Chryseobacterium*	0	1	3	0	4
						*Taibaiella*	0	0	1	0	0
Alphaproteobacteria	1	1	1	0	1	*Phyllobacterium*	1	0	0	0	0
						*Ensifer*	0	1	0	0	0
						*Sphingomonas*	0	0	1	0	0
						*Paracoccus*	0	0	0	0	1
Betaproteobacteria	10	11	13	2	5	*Variovorax*	10	11	13	1	4
						*Massilia*	0	0	0	1	1
Gammaproteobacteria	60	14	6	0	10	*Pseudomonas*	58	10	0	0	2
						*Pseudoxanthomonas*	0	0	1	0	0
						*Stenotrophomonas*	2	0	2	0	0
						*Acinetobacter*	0	2	1	0	0
						*Xanthomonas*	0	2	0	0	3
						*Luteibacter*	0	0	1	0	0
						*Lysobacter*	0	0	1	0	5
Total *n* of isolates	80	70	69	75	77		80	70	69	75	77

The table indicates the number of isolates classified at the Phylum/Class and at the Genus levels

### Plant Growth Promotion Potential within the Bacteria Collection Established from *A. spinosa* Root System, Bulk Soil, and Residuesphere

Some of the genera detected in the established bacteria collection (e.g., *Arthrobacter, Microbacterium*) include species that are well adapted to the extreme conditions of dry soil where argan trees thrive and can act as plant growth promoters [75, 76]. One strain from each ITS groups (*n* = 219) was tested in vitro for a set of direct and indirect activities related to plant growth promotion (PGP). The investigation of the PGP potential of the isolates focused in particular on the screening of (i) the biofertilization capacity (i.e., phosphate solubilization activity and the production of siderophores and ammonia), (ii) the occurrence of traits related to biocontrol activities, also important for organic matter degradation in the residuesphere (i.e., protease and esterase activities), and (iii) the production of exopolysaccharides that can ameliorate the water-holding capacity of soil and promote the stability of soil aggregates, particularly relevant in arid environments [77]. The results of PGP-related trait assays (reported in detail in Supplementary Table 3) showed that the highest numbers of positive isolates were observed in the *Bacillaceae*, *Flavobacteriaceae*, *Micrococcaceae*, *Streptomycetaceae*, *Comamonadaceae, Xanthomonadaceae*, and *Pseudomonadaceae* families (Fig. 4d, Supplementary Table 3). The bacteria affiliated to these taxa were widely reported as plant growth–promoting bacteria selected in the root endosphere and/or rhizosphere by a range of different plant species under extreme conditions such as soil salinity and water scarcity [14, 16]. Extremophilic EPS-producing bacteria isolated from desert and saline systems were able to produce biosurfactants/bioemulsifiers that under controlled laboratory conditions proved to increase water retention of a sandy soil [78], an aspect of great interest to reverse desertification. However, in this study, EPS production was detected only in one strain, *Phyllobacterium ifriqiyense*-A-8E16 (Supplementary Table 3), an endophyte previously isolated from the root nodule of two leguminous plant species in Southern Tunisia [79]. Indeed, EPS-producing strains can be more easily identified through specific isolation media [80, 81]. Ammonia production was a common trait in the bacteria collection, with 199 positive isolates over the tested 219, in accordance with previous reports of bacteria associated to the halophyte *Salicornia* [43]. A high number of positive isolates was detected also for siderophore production (*n* = 61) while those showing phosphate solubilization capacity (*n* = 27) were less abundant. Protease and esterase activities were traits widely spread in the bacteria collection (*n* = 109 and *n* = 39, respectively; Supplementary Table 3). These are cell wall–degrading enzymes with a potential role in biocontrol (a PGP indirect mechanism), but they are also involved in the degradation of the organic matter and are in fact abundant among the strains isolated from the residuesphere, the niche where plant cell material is primarily degraded. Likewise, Egamberdieva and coauthors [82] found a higher abundance of cell wall–degrading enzymes among bacteria isolated from hydrochar-supplemented soil compared to the control one. Interestingly, in their work, the authors could detect a significant increase in soybean growth and a higher diversity of soybean rhizospheric bacteria exclusively in the soil subjected to the hydrochar addition, suggesting a combined plant growth promotion effect due to nutrient supply and the stimulation of a more effective rhizospheric community [82].

The results of the PGP assays were used to calculate for each isolate a “PGP score”, reporting the total number of positive activities. Isolates that did not harbor PGP traits were detected in all the fractions. Only in the residuesphere, the 100% of the isolates displayed at least one PGP trait, demonstrating that this niche could be particularly important for the selection of plant beneficial bacteria (Fig. 4e). None of the tested bacteria displayed the complete set of PGP activities (Supplementary Table 2), while most of the isolates showed a “PGP score” comprised between 1 and 3 (Fig. 4e), and they were present in all the sub-collections obtained from the root, soils, and residuesphere. Noteworthy, most of the bacterial strains showing a “PGP score” of 4 were isolated from the residuesphere fraction (*n* = 7) and were not present within the root endosphere and bulk soil sub-collections. We did not detect a clear fractioning of the PGP potential associated to the cultivable bacteria isolated from *A. spinosa* in a specific ecological niche; however, the fact that the multivalent isolates with the highest PGP score were mostly identified in the residuesphere led us to hypothesize that these bacteria play a central role in the argan litter effect [5], as recently suggested also for the addition of biochar [82]. Previous evidences showed that the residuesphere is enriched in ammonia-oxidizing bacteria (AOB) able to increase nitrogen bioavailability for the plant, a critical aspect in arid environments [83]. In addition, the promising results shown by residuesphere-dwelling bacteria can be also related to the fact that our study included the screening of PGP traits like esterase and protease activities, which are among the key traits involved in the organic matter degradation typically played by microbial communities in the litter [9]. We propose that the high PGP potential observed among the residuesphere bacterial isolates cooperates to the increased nutrient bioavailability determined by the addition of argan litter to the soil, a common practice in South Western Morocco.

## Conclusions

The xerophyte microbiome is a still-overlooked source of microbial resources potentially useful for environmental and agriculture biotechnology application. Currently, no reports are available concerning the bacterial communities associated to the tree *Argania spinosa* L. Skeels although UNESCO has defined their forest in South Morocco as a biosphere reserve [26]. The 16S rRNA Illumina sequencing dataset showed that the root system portions of *A. spinosa* hosted different bacterial communities according to their degree of association with the plants. Trends of beta-diversity could be observed both considering the entire bacterial communities described by 16S rRNA Illumina sequencing and the cultivable fraction obtained from the root endosphere (E), the root-associated soils (R and SSR), the bulk soil (B), and the residuesphere (Re). Our data showed that the root system of *A. spinosa* and the residuesphere developed from its litter are associated to a high number of bacterial taxa endowed with direct and indirect plant growth promotion activities. In particular, we report here that the residuesphere was colonized by a cultivable bacterial community clearly distinguished from the other analyzed samples that, according to the performed in vitro screening, is involved in organic matter decomposition and showed the highest PGP potential. These bacterial strains, in concert with the argan root system, could contribute to the litter effect previously described for this tree species [5], possibly providing a scientific interpretation behind the traditional use of argan litter by Moroccan farmers as soil amendment.

## Electronic supplementary material

Fig. S1 Rarefaction curve of the 16S rRNA Illumina libraries Rarefaction curves were calculated for each sample. E: root endosphere; R: rhizosphere; SSR: root surrounding soil; B: bulk soil; Re: residuesphere (PNG 146 kb)

High resolution image (TIF 1075 kb)

Fig. S2 Cultivable bacteria abundance. Bacteria abundance is expressed as colony forming unit (cfu)/g of environmental sample used for isolation: root tissue for the fraction E (endosphere), soil for fractions R (rhizosphere), SSR (root surrounding soil) and B (bulk soil), and litter for the fraction Re (residuesphere). In each bar, the standard deviation refers to technical replicates (*n* = 3) (PNG 51 kb)

High resolution image (TIF 760 kb)

Table S1 (DOCX 58 kb)
