# Supplementary figures and images for: Unveiling the Microbiota Diversity of the Xerophyte Argania spinosa L. Skeels Root System and Residuesphere
Source: Microb Ecol. 2020 Jun 25;80(4):822–36. doi: 10.1007/s00248-020-01543-4 (PMC7550381; doi:10.1007/s00248-020-01543-4)

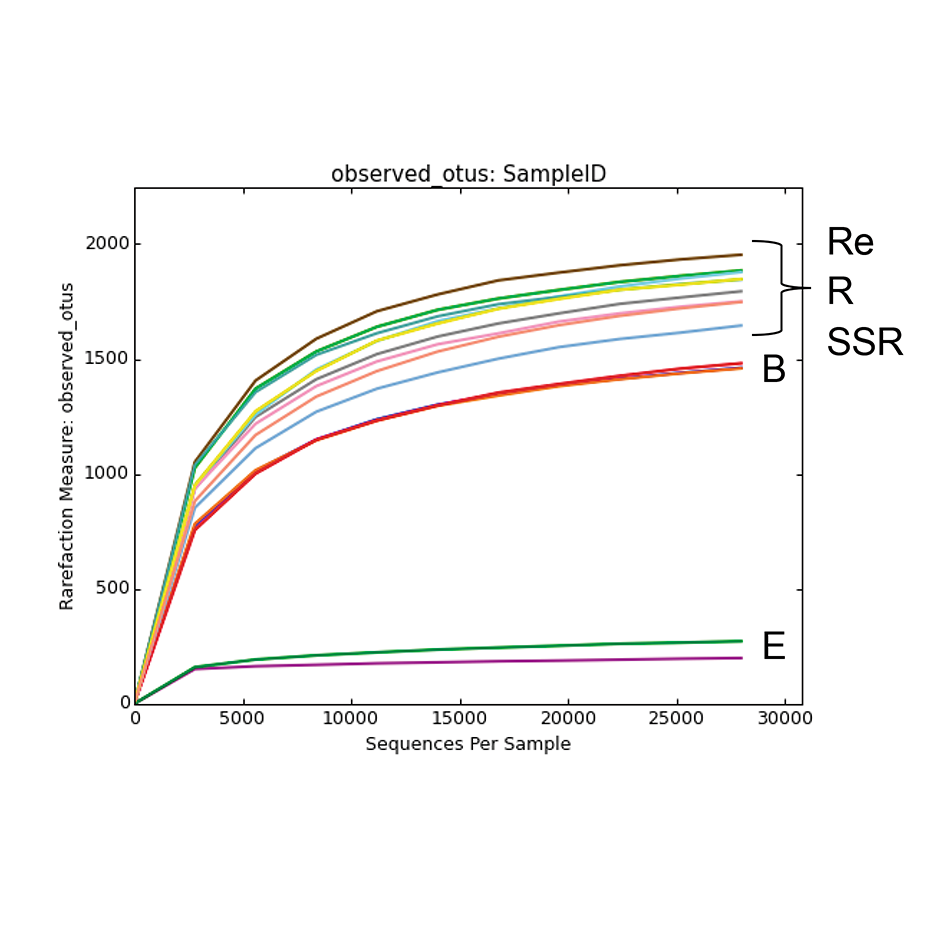

Supplement: Supplementary file 1 — Rarefaction curve of the 16S rRNA Illumina libraries Rarefaction curves were calculated for each sample. E: root endosphere; R: rhizosphere; SSR: root surrounding soil; B: bulk soil; Re: residuesphere (PNG 146 kb) [file 248_2020_1543_Fig5_ESM.png]

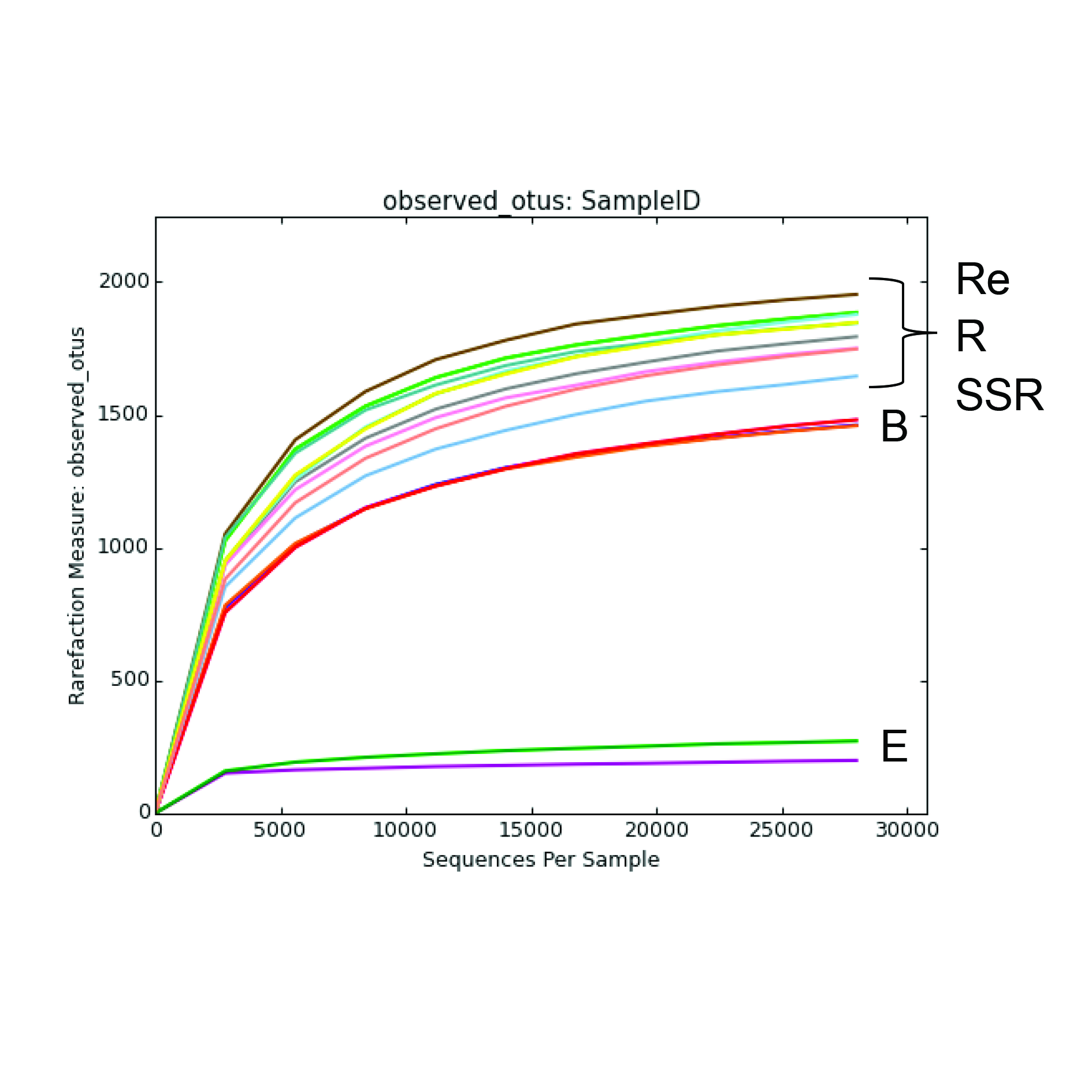

Supplement: Supplementary file 2 — High resolution image (TIF 1075 kb) [file 248_2020_1543_MOESM1_ESM.tif]

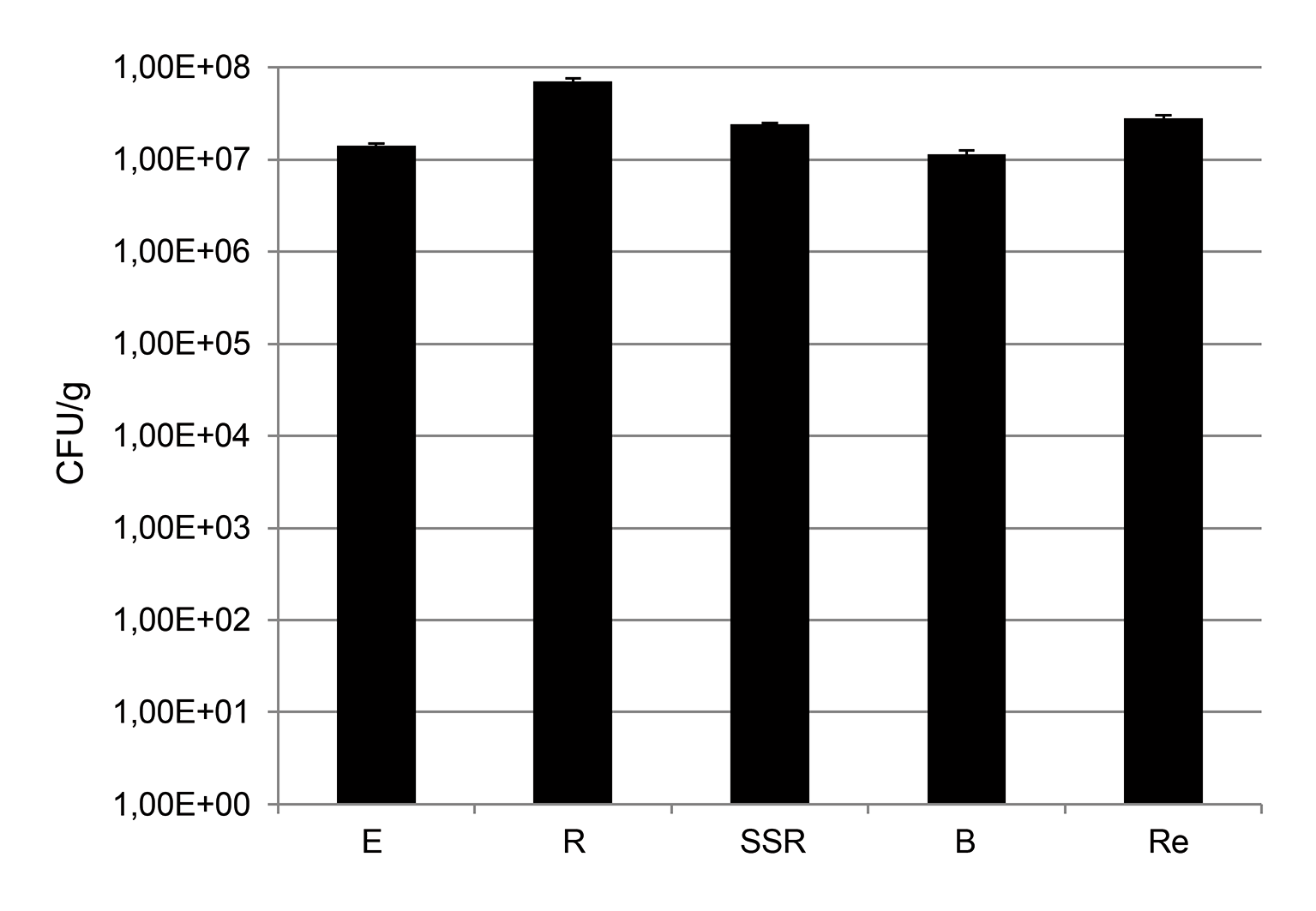

Supplement: Supplementary file 3 — Cultivable bacteria abundance. Bacteria abundance is expressed as colony forming unit (cfu)/g of environmental sample used for isolation: root tissue for the fraction E (endosphere), soil for fractions R (rhizosphere), SSR (root surrounding soil) and B (bulk soil), and litter for the fraction Re (residuesphere). In each bar, the standard deviation refers to technical replicates (n = 3) (PNG 51 kb) [file 248_2020_1543_Fig6_ESM.png]

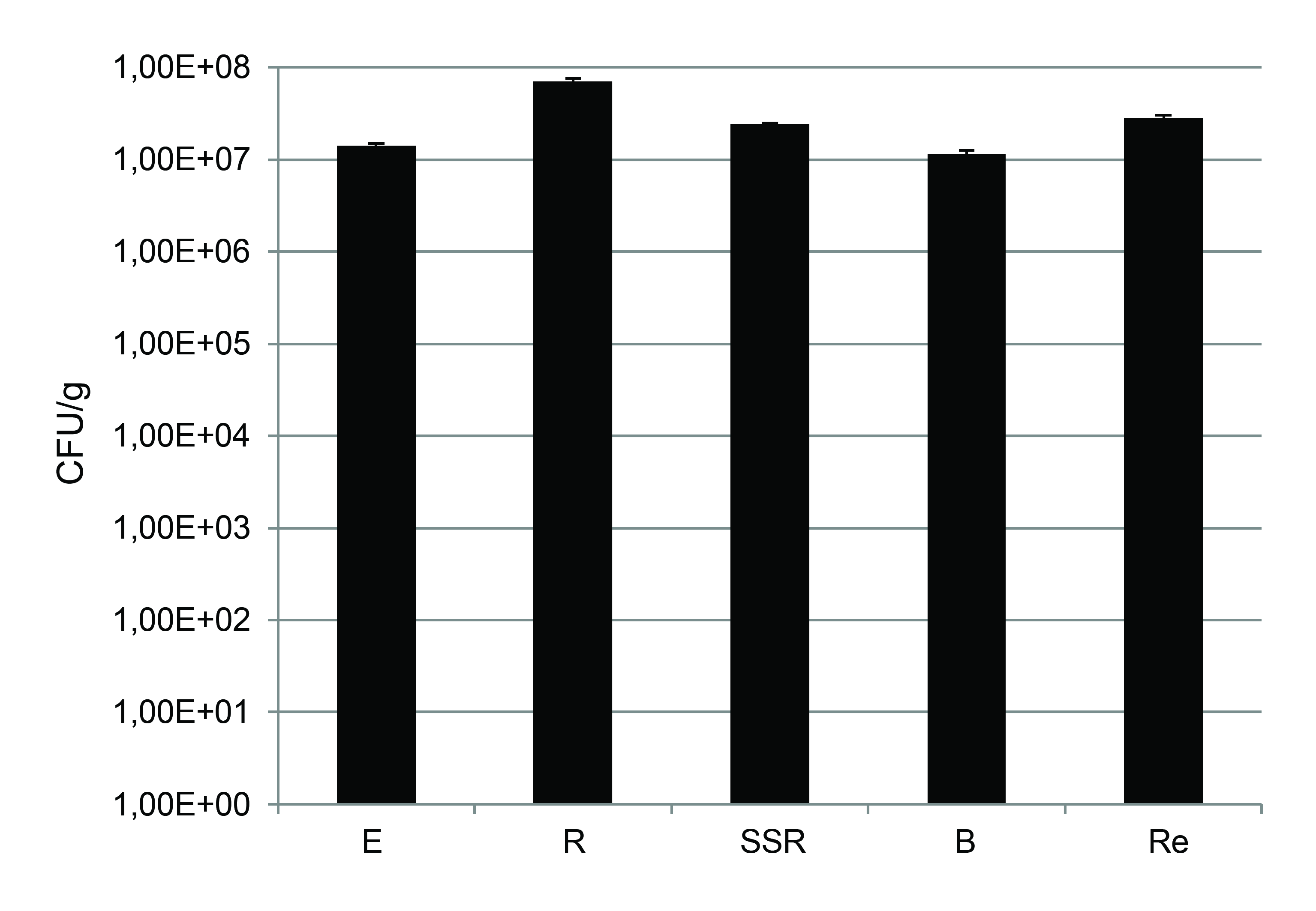

Supplement: Supplementary file 4 — High resolution image (TIF 760 kb) [file 248_2020_1543_MOESM2_ESM.tif]
