# Supplementary material for: Unveiling the Microbiota Diversity of the Xerophyte Argania spinosa L. Skeels Root System and Residuesphere
Source: Microb Ecol. 2020 Jun 25;80(4):822–36. doi: 10.1007/s00248-020-01543-4 (PMC7550381; doi:10.1007/s00248-020-01543-4)
Supplement: Supplementary file 5 — (DOCX 58 kb) [file 248_2020_1543_MOESM3_ESM.docx]

**Supplementary Table 1. Distribution of the taxonomic groups (at the Class level) according to the phylogenetic affiliation of the OTU_97_ detected by 16S rRNA Illumina sequencing.** The relative abundance of each group is expressed as percentage over the total bacterial community, E= endosphere; R= rhizosphere; SSR= root-surrounding soil; B= bulk soil; Re= residuesphere.

| **Phylum** | **Class** | **E1** | **E2** | **R1** | **R2** | **R3** | **SSR1** | **SSR2** | **SSR3** | **B1** | **B2** | **B3** | **Re1** | **Re2** | **Re3** |
| --- | --- | --- | --- | --- | --- | --- | --- | --- | --- | --- | --- | --- | --- | --- | --- |
| Other | Other | 0,0000 | 0,0000 | 0,1388 | 0,2883 | 0,4698 | 0,0854 | 0,1708 | 0,3702 | 0,1317 | 0,1317 | 0,1424 | 0,0890 | 0,1424 | 0,1459 |
| Acidobacteria | Acidobacteria-5 | 0,0000 | 0,0000 | 0,0107 | 0,0783 | 0,0925 | 0,0498 | 0,1424 | 0,1673 | 0,0214 | 0,0214 | 0,0142 | 0,0249 | 0,0463 | 0,0356 |
| Acidobacteria | Acidobacteria-6 | 0,0036 | 0,8898 | 3,6269 | 4,4241 | 7,8623 | 7,3071 | 11,3895 | 9,6882 | 4,9900 | 4,5273 | 5,3815 | 6,0507 | 7,0829 | 5,0114 |
| Acidobacteria | Acidobacteriia | 0,0000 | 0,0000 | 0,0142 | 0,0071 | 0,0178 | 0,0392 | 0,0178 | 0,0320 | 0,0036 | 0,0071 | 0,0036 | 0,0285 | 0,0285 | 0,0285 |
| Acidobacteria | Solibacteres | 0,0000 | 0,0000 | 1,0962 | 0,6264 | 1,4878 | 1,3454 | 0,7866 | 1,8152 | 2,6303 | 2,1355 | 2,0216 | 1,2208 | 1,2564 | 1,5447 |
| Acidobacteria | Sva0725 | 0,0000 | 0,0000 | 0,2598 | 0,3951 | 0,2669 | 0,2385 | 0,2100 | 1,5483 | 0,8791 | 0,9396 | 0,6691 | 0,2919 | 1,1318 | 0,3737 |
| Acidobacteria | [Chloracidobacteria] | 0,0000 | 0,0000 | 1,5874 | 4,7373 | 1,7262 | 3,1748 | 6,7091 | 1,6764 | 6,8871 | 7,4459 | 10,0157 | 2,9114 | 4,0504 | 3,3279 |
| Acidobacteria | iii1-8 | 0,0000 | 0,0000 | 0,0783 | 0,1068 | 0,9468 | 0,2598 | 0,5659 | 1,3312 | 0,4378 | 0,3274 | 0,4663 | 0,2705 | 0,3595 | 0,3132 |
| Actinobacteria | Acidimicrobiia | 0,0320 | 0,6869 | 1,4415 | 1,4557 | 1,5874 | 1,1639 | 1,3383 | 1,9896 | 1,0962 | 1,1639 | 1,2884 | 1,2315 | 1,0251 | 0,9966 |
| Actinobacteria | Actinobacteria | 15,7496 | 15,9240 | 25,2954 | 15,5182 | 10,6243 | 17,4829 | 7,1861 | 7,6950 | 9,3181 | 12,1405 | 8,3749 | 15,8350 | 13,3079 | 18,9813 |
| Actinobacteria | MB-A2-108 | 0,0000 | 0,0000 | 0,1566 | 0,8862 | 1,1283 | 0,2242 | 1,1496 | 1,7511 | 0,6585 | 0,5019 | 0,5979 | 0,0961 | 0,4449 | 0,2349 |
| Actinobacteria | Nitriliruptoria | 0,0000 | 0,0000 | 0,0498 | 0,1175 | 0,4022 | 0,0819 | 0,0819 | 0,8649 | 0,0854 | 0,0925 | 0,0819 | 0,0605 | 0,0783 | 0,0747 |
| Actinobacteria | Rubrobacteria | 0,0000 | 0,0000 | 4,0575 | 9,3572 | 7,2822 | 2,9328 | 8,3144 | 10,0085 | 7,0010 | 6,4280 | 5,8763 | 1,5767 | 4,6519 | 2,2494 |
| Actinobacteria | Thermoleophilia | 0,0071 | 0,0000 | 7,3534 | 6,3283 | 3,4809 | 5,1680 | 6,2785 | 4,1643 | 3,0681 | 3,6589 | 3,8297 | 3,5877 | 3,1819 | 2,5520 |
| Armatimonadetes | Armatimonadia | 0,0000 | 0,0000 | 0,0392 | 0,1175 | 0,0427 | 0,0747 | 0,0498 | 0,0498 | 0,3986 | 0,4129 | 0,3915 | 0,0819 | 0,1673 | 0,4271 |
| Armatimonadetes | [Fimbriimonadia] | 0,0000 | 0,0000 | 0,0000 | 0,0178 | 0,0178 | 0,0036 | 0,0107 | 0,0036 | 0,0142 | 0,0107 | 0,0285 | 0,0000 | 0,0036 | 0,0498 |
| Bacteroidetes | At12OctB3 | 0,0000 | 0,0000 | 0,0071 | 0,0036 | 0,0071 | 0,0071 | 0,0142 | 0,0071 | 0,0320 | 0,0071 | 0,0712 | 0,0142 | 0,0036 | 0,0036 |
| Bacteroidetes | Cytophagia | 0,8115 | 0,9752 | 2,1498 | 2,2138 | 1,9860 | 2,7370 | 1,9967 | 1,3739 | 6,2856 | 4,9722 | 4,3601 | 3,5023 | 3,0218 | 3,3528 |
| Bacteroidetes | Flavobacteriia | 11,8522 | 1,3525 | 1,2635 | 1,6088 | 0,6442 | 0,6442 | 0,3880 | 0,6086 | 0,0214 | 0,0071 | 0,0463 | 1,9825 | 0,7724 | 0,5268 |
| Bacteroidetes | Sphingobacteriia | 30,8478 | 0,1602 | 0,5196 | 0,5196 | 0,3915 | 0,7403 | 0,6122 | 0,6727 | 0,1602 | 0,1068 | 0,1851 | 1,6515 | 0,6905 | 0,8293 |
| Bacteroidetes | [Rhodothermi] | 0,0000 | 0,0000 | 0,0036 | 0,0000 | 0,7652 | 0,0000 | 0,0036 | 1,3667 | 0,0000 | 0,0036 | 0,0071 | 0,0000 | 0,0036 | 0,0249 |
| Bacteroidetes | [Saprospirae] | 0,4698 | 4,8405 | 1,6372 | 1,4379 | 2,9043 | 2,4310 | 1,3917 | 2,2565 | 2,2850 | 1,8223 | 2,1071 | 3,7265 | 2,7442 | 3,0787 |
| Chlorobi | Unc. Chlorobi | 0,0000 | 0,0000 | 0,0285 | 0,0178 | 0,0107 | 0,0036 | 0,0569 | 0,0071 | 0,0142 | 0,0214 | 0,0285 | 0,0569 | 0,0285 | 0,0356 |
| Chlorobi | OPB56 | 0,0000 | 0,0000 | 0,0214 | 0,0392 | 0,1495 | 0,0534 | 0,0676 | 0,0178 | 0,0534 | 0,0392 | 0,0676 | 0,0712 | 0,0819 | 0,0498 |
| Chloroflexi | Unc. Chloroflexi | 0,0000 | 0,0000 | 0,0641 | 0,1424 | 0,1103 | 0,0819 | 0,0605 | 0,0997 | 0,0783 | 0,0747 | 0,0712 | 0,0142 | 0,0819 | 0,0854 |
| Chloroflexi | Anaerolineae | 0,0036 | 0,0000 | 0,1388 | 0,2064 | 0,2136 | 0,1068 | 0,1246 | 0,2563 | 0,1068 | 0,1459 | 0,1032 | 0,0890 | 0,1815 | 0,2242 |
| Chloroflexi | C0119 | 0,0000 | 0,0000 | 0,0534 | 0,0676 | 0,0320 | 0,0142 | 0,0107 | 0,0071 | 0,0427 | 0,0392 | 0,0534 | 0,0107 | 0,0320 | 0,0214 |
| Chloroflexi | Chloroflexi | 0,0000 | 0,0000 | 0,5552 | 1,5483 | 0,4841 | 0,6229 | 0,6727 | 0,4912 | 1,6088 | 1,4166 | 1,3240 | 0,2349 | 0,7581 | 0,7190 |
| Chloroflexi | Ellin6529 | 0,0000 | 0,0000 | 0,5481 | 0,8507 | 0,3310 | 0,4734 | 0,5979 | 0,3203 | 0,1673 | 0,2064 | 0,1958 | 0,2527 | 0,3559 | 0,3061 |
| Chloroflexi | Gitt-GS-136 | 0,0000 | 0,0000 | 0,1388 | 0,1637 | 0,7937 | 0,1068 | 0,1495 | 0,6763 | 0,0142 | 0,0249 | 0,0249 | 0,0997 | 0,0641 | 0,0676 |
| Chloroflexi | S085 | 0,0000 | 0,0000 | 0,3097 | 0,5196 | 0,3595 | 0,2171 | 0,3630 | 0,7261 | 0,1388 | 0,1210 | 0,1068 | 0,1566 | 0,1673 | 0,1317 |
| Chloroflexi | SAR202 | 0,0000 | 0,0000 | 0,0000 | 0,0392 | 0,0569 | 0,0000 | 0,0641 | 0,0534 | 0,0036 | 0,0107 | 0,0000 | 0,0000 | 0,0071 | 0,0000 |
| Chloroflexi | TK10 | 0,0000 | 0,0000 | 0,8008 | 1,5020 | 0,6371 | 0,5766 | 1,4949 | 0,8756 | 0,6051 | 0,6798 | 0,7546 | 0,4378 | 0,7332 | 0,4556 |
| Chloroflexi | TK17 | 0,0000 | 0,0000 | 0,1602 | 0,1851 | 0,3524 | 0,1495 | 0,1530 | 0,5125 | 0,0285 | 0,0427 | 0,0356 | 0,1780 | 0,0605 | 0,0676 |
| Chloroflexi | Thermomicrobia | 0,0036 | 0,0036 | 2,5484 | 3,5841 | 2,6125 | 1,5554 | 1,9576 | 2,7691 | 1,5910 | 1,5874 | 1,7725 | 1,0678 | 1,8188 | 1,7511 |
| Cyanobacteria | Unc. Cyanobacteria | 0,0000 | 0,0000 | 0,0000 | 0,0000 | 0,0000 | 0,0000 | 0,0000 | 0,0000 | 0,3061 | 0,7154 | 0,5161 | 0,0000 | 0,0000 | 0,0000 |
| Cyanobacteria | 4C0d-2 | 0,0000 | 0,0000 | 0,0000 | 0,0107 | 0,0463 | 0,0214 | 0,1210 | 0,0605 | 0,0356 | 0,0107 | 0,0107 | 0,0036 | 0,0142 | 0,0000 |
| Cyanobacteria | Chloroplast | 0,1602 | 12,0907 | 0,0819 | 0,0427 | 0,2420 | 0,0819 | 0,0036 | 0,0071 | 0,3132 | 0,8257 | 0,2990 | 0,0107 | 0,0854 | 0,0107 |
| Cyanobacteria | Nostocophycideae | 0,0000 | 0,0000 | 0,0000 | 0,0000 | 0,0000 | 0,0000 | 0,0000 | 0,0000 | 0,0498 | 0,4235 | 0,0392 | 0,0000 | 0,0000 | 0,0000 |
| Cyanobacteria | Oscillatoriophycideae | 0,0000 | 0,0000 | 0,0000 | 0,0000 | 0,0000 | 0,0000 | 0,0000 | 0,0000 | 0,4413 | 0,9823 | 0,5944 | 0,0000 | 0,0071 | 0,0000 |
| Cyanobacteria | Synechococcophycideae | 0,0000 | 0,0000 | 0,0000 | 0,0000 | 0,0000 | 0,0000 | 0,0000 | 0,0000 | 0,1139 | 0,3666 | 0,0997 | 0,0000 | 0,0000 | 0,0036 |
| Elusimicrobia | Elusimicrobia | 0,0000 | 0,0000 | 0,0142 | 0,0036 | 0,0427 | 0,0178 | 0,0000 | 0,0356 | 0,0107 | 0,0071 | 0,0214 | 0,0107 | 0,0142 | 0,0249 |
| FBP | Unc. FBP | 0,0000 | 0,0000 | 0,0178 | 0,0747 | 0,0427 | 0,0071 | 0,0285 | 0,0605 | 0,3951 | 0,2278 | 0,2705 | 0,0320 | 0,0392 | 0,1281 |
| Fibrobacteres | Fibrobacteria | 0,0000 | 0,1353 | 0,0249 | 0,0214 | 0,0569 | 0,0320 | 0,0498 | 0,0747 | 0,0356 | 0,0569 | 0,0249 | 0,0463 | 0,0249 | 0,0000 |
| Firmicutes | Bacilli | 0,0000 | 0,2634 | 2,7157 | 1,0073 | 2,8723 | 1,1817 | 1,2315 | 2,3064 | 1,3703 | 0,9254 | 0,5303 | 0,3559 | 1,0322 | 0,4164 |
| Gemmatimonadetes | Gemm-1 | 0,0000 | 0,0000 | 0,4058 | 0,7937 | 0,2705 | 0,5873 | 1,1425 | 0,1459 | 1,0856 | 0,8044 | 0,8079 | 0,3880 | 0,6371 | 0,6549 |
| Gemmatimonadetes | Gemm-2 | 0,0000 | 0,0000 | 0,0605 | 0,1495 | 0,1317 | 0,0783 | 0,3274 | 0,3524 | 0,0071 | 0,0071 | 0,0107 | 0,0712 | 0,1281 | 0,0392 |
| Gemmatimonadetes | Gemm-3 | 0,0000 | 0,0000 | 0,8222 | 1,8508 | 4,8690 | 1,0037 | 0,9859 | 4,5736 | 1,0784 | 0,8613 | 1,0464 | 1,6622 | 1,2671 | 1,0144 |
| Gemmatimonadetes | Gemm-5 | 0,0000 | 0,0000 | 0,6122 | 1,2030 | 1,9967 | 0,9040 | 2,7264 | 2,7620 | 0,3310 | 0,2563 | 0,3559 | 0,9859 | 1,1140 | 0,6371 |
| Gemmatimonadetes | Gemmatimonadetes | 0,0000 | 0,0000 | 0,7617 | 2,1640 | 2,1640 | 1,3810 | 3,7799 | 2,1854 | 1,3561 | 1,3383 | 1,8401 | 1,2208 | 2,0501 | 1,3739 |
| Nitrospirae | Nitrospira | 0,0000 | 0,0000 | 0,2171 | 0,3808 | 0,4591 | 0,4164 | 1,2244 | 0,5873 | 0,7581 | 0,5766 | 0,5232 | 0,2883 | 0,5552 | 0,4912 |
| OD1 | Unc. OD1 | 0,0000 | 0,0000 | 0,0000 | 0,0000 | 0,0285 | 0,0000 | 0,0000 | 0,0498 | 0,0000 | 0,0000 | 0,0000 | 0,0000 | 0,0000 | 0,0000 |
| Planctomycetes | Phycisphaerae | 0,0036 | 0,1210 | 1,9149 | 2,4559 | 3,9223 | 3,8546 | 4,0504 | 3,0253 | 7,5491 | 7,6025 | 10,5638 | 5,4278 | 4,5985 | 4,5629 |
| Planctomycetes | Planctomycetia | 0,0071 | 0,6335 | 2,7335 | 2,1249 | 3,3919 | 4,1714 | 2,8260 | 4,1038 | 2,9470 | 3,6553 | 3,2247 | 3,9329 | 3,7407 | 3,6945 |
| Proteobacteria | Alphaproteobacteria | 5,5488 | 13,9415 | 21,7896 | 16,5860 | 15,4221 | 19,7110 | 11,0585 | 12,6993 | 21,3304 | 21,8003 | 19,6078 | 20,1666 | 20,0420 | 24,5302 |
| Proteobacteria | Betaproteobacteria | 2,5982 | 26,4664 | 3,0609 | 4,0824 | 4,4206 | 4,5487 | 6,9334 | 4,3672 | 2,1818 | 1,8472 | 1,9505 | 4,4312 | 4,9188 | 4,1429 |
| Proteobacteria | Deltaproteobacteria | 0,0427 | 2,8011 | 3,0431 | 3,7550 | 3,0681 | 4,4348 | 4,1856 | 2,2957 | 1,8935 | 1,7298 | 1,5589 | 4,9046 | 4,3565 | 4,3423 |
| Proteobacteria | Gammaproteobacteria | 31,4885 | 17,9207 | 4,0006 | 2,1320 | 2,9470 | 4,8156 | 2,2921 | 1,6301 | 1,0642 | 0,9290 | 0,8756 | 5,3780 | 3,9614 | 3,9970 |
| TM7 | Unc. TM7 | 0,0000 | 0,0000 | 0,0107 | 0,0000 | 0,4235 | 0,0107 | 0,0000 | 0,0178 | 0,0000 | 0,0071 | 0,0000 | 0,0000 | 0,0000 | 0,0000 |
| TM7 | SC3 | 0,0000 | 0,0000 | 0,0000 | 0,0000 | 0,0997 | 0,0000 | 0,0000 | 0,1495 | 0,0036 | 0,0036 | 0,0036 | 0,0000 | 0,0000 | 0,0036 |
| TM7 | TM7-1 | 0,0000 | 0,0000 | 0,1246 | 0,0641 | 0,1851 | 0,1246 | 0,1530 | 0,1103 | 0,2598 | 0,0178 | 0,0819 | 0,0925 | 0,1246 | 0,0356 |
| TM7 | TM7-3 | 0,0036 | 0,0000 | 0,1851 | 0,4841 | 0,1103 | 0,1459 | 0,2100 | 0,0569 | 0,0000 | 0,0071 | 0,0178 | 0,1424 | 0,1175 | 0,0890 |
| Tenericutes | Mollicutes | 0,0000 | 0,0000 | 0,0036 | 0,0071 | 0,0000 | 0,0071 | 0,0036 | 0,0000 | 0,0676 | 0,0285 | 0,0498 | 0,0000 | 0,0285 | 0,0000 |
| Verrucomicrobia | Opitutae | 0,0214 | 0,2527 | 0,3203 | 0,3524 | 0,5517 | 0,4734 | 0,4520 | 0,4556 | 0,2883 | 0,2776 | 0,4129 | 0,7368 | 0,5730 | 0,3381 |
| Verrucomicrobia | Verrucomicrobiae | 0,3346 | 0,2954 | 0,3097 | 0,2207 | 0,7047 | 0,2883 | 0,1708 | 0,2100 | 0,0427 | 0,0285 | 0,0285 | 0,6549 | 0,3168 | 0,2207 |
| Verrucomicrobia | [Pedosphaerae] | 0,0000 | 0,0000 | 0,2812 | 0,6229 | 0,6763 | 0,6407 | 0,8827 | 0,8471 | 0,5446 | 0,3630 | 0,5552 | 0,6656 | 0,6620 | 0,4129 |
| Verrucomicrobia | [Spartobacteria] | 0,0107 | 0,2456 | 0,3132 | 0,2812 | 0,3844 | 0,8862 | 0,5374 | 0,3097 | 3,2816 | 2,0430 | 3,5806 | 1,4771 | 1,0037 | 0,7332 |
| WS2 | SHA-109 | 0,0000 | 0,0000 | 0,0214 | 0,0178 | 0,0142 | 0,0214 | 0,0427 | 0,0249 | 0,0000 | 0,0000 | 0,0000 | 0,0107 | 0,0214 | 0,0214 |
| [Thermi] | Deinococci | 0,0000 | 0,0000 | 0,0000 | 0,0000 | 0,1780 | 0,0000 | 0,0000 | 0,2420 | 0,0000 | 0,0000 | 0,0000 | 0,0000 | 0,0000 | 0,0036 |
|  |  | 100 | 100 | 100 | 100 | 100 | 100 | 100 | 100 | 100 | 100 | 100 | 100 | 100 | 100 |

**Supplementary Table 2. Phylogenetic identification of the bacterial isolates.** List of the bacterial strains isolated from each fraction, identified by 16S rRNA gene sequencing (one representative strain for each ITS group has been sequenced) and blast search in the NCBI public database. The column ‘n° isolates/ITS’ reports the number of isolates corresponding to each representative of the ITS groups. The accession number of the closest described relative species in the NCBI public database is reported in the column ‘acc. n° (NCBI)’. The last column indicates the size of the 16S rRNA amplicon of each isolate (on the left) in comparison with that or the closest described relative in NCBI (on the right).

| **Fraction** | **Isolate** | **n° isolates/ ITS** | **Closest described relative (BLAST)** | **acc.n° (NCBI)** | **Id %** | **base** |
| --- | --- | --- | --- | --- | --- | --- |
| E | A-8E29 | 1 | *Arthrobacter polychromogenes* | KR085878 | 99 | 886/892 |
| E | A-8E62 | 3 | *Arthrobacter oryzae* | KR233762 | 99 | 939/950 |
| E | A-8E73 | 1 | *Bacillus simplex* | CP017704 | 100 | 999/999 |
| E | A-8E26 | 4 | *Flavobacterium piscis* | NR_133746 | 98 | 883/900 |
| E | A-8E16 | 1 | *Phyllobacterium ifriqiyense* | KF844047 | 99 | 898/899 |
| E | A-8E1 | 56 | *Pseudomonas fluorescens* | KR187014 | 99 | 886/892 |
| E | A-8E31 | 2 | *Pseudomonas frederiksbergensis* | HF584798 | 100 | 849/849 |
| E | A-8E54 | 2 | *Stenotrophomonas tumulicola* | LC066089 | 100 | 879/879 |
| E | A-8E2 | 8 | *Variovorax paradoxus* | KU258286 | 99 | 895/896 |
| E | A-8E57 | 1 | *Variovorax paradoxus* | MF101074 | 99 | 907/914 |
| E | A-8E74 | 1 | *Variovorax paradoxus* | MF101074 | 99 | 846/848 |
| R | A-8R65 | 2 | *Acinetobacter johnsonii* | KP236312 | 99 | 699/701 |
| R | A-8R7 | 1 | *Arthrobacter pascens* | KF515608 | 99 | 899/900 |
| R | A-8R9 | 2 | *Arthrobacter polychromogenes* | KR085878 | 99 | 844/850 |
| R | A-8R10 | 1 | *Paenarthrobacter nitroguajacolicus* | KX036598.1 | 100 | 949/949 |
| R | A-8R12 | 3 | *Arthrobacter oxydans* | LN774368 | 100 | 898/898 |
| R | A-8R14 | 4 | *Paenarthrobacter nitroguajacolicus* | MF796802 | 100 | 549/549 |
| R | A-8R21 | 2 | *Arthrobacter aurescens* | JX293327 | 100 | 849/849 |
| R | A-8R22 | 1 | *Arthrobacter aurescens* | JX293327 | 99 | 783/786 |
| R | A-8R37 | 1 | *Arthrobacter oxydans* | FR877676 | 99 | 696/700 |
| R | A-8R38 | 2 | *Paenarthrobacter nitroguajacolicus* | KX036598 | 100 | 899/899 |
| R | A-8R44 | 2 | *Arthrobacter oxydans* | JF496330 | 99 | 798/799 |
| R | A-8R54 | 1 | *Arthrobacter oxydans* | JF496330 | 100 | 799/799 |
| R | A-8R60 | 3 | *Arthrobacter pascens* | KY933458 | 99 | 695/700 |
| R | A-8R69 | 1 | *Arthrobacter pascens* | KC934839 | 99 | 698/702 |
| R | A-8R4 | 1 | *Brevibacterium frigoritolerans* | KU877654 | 100 | 798/798 |
| R | A-8R20 | 1 | *Bacillus thuringiensis* | KM269287 | 99 | 748/749 |
| R | A-8R43A | 1 | *Bacillus deserti* | KT720271 | 99 | 844/847 |
| R | A-8R5 | 1 | *Paenibacillus xylanilyticus* | KT003257 | 97 | 624/645 |
| R | A-8R2 | 1 | *Chryseobacterium arachidis* | NR_133723 | 98 | 877/899 |
| R | A-8R30 | 1 | *Sinorhizobium meliloti* | KC172024 | 100 | 849/849 |
| R | A-8R1 | 2 | *Flavobacterium aquidurense* | NR_042470 | 98 | 982/999 |
| R | A-8R13 | 1 | *Flavobacterium johnsoniae* | AM921621 | 98 | 783/799 |
| R | A-8R23 | 1 | *Flavobacterium pectinovorum* | AM934674 | 97 | 767/793 |
| R | A-8R28 | 1 | *Flavobacterium saccharophilum* | KR085800 | 98 | 590/601 |
| R | A-8R32 | 1 | *Flavobacterium pectinovorum* | NR_104717 | 97 | 756/789 |
| R | A-8R52 | 1 | *Flavobacterium saccharophilum* | KR085800 | 97 | 763/784 |
| R | A-8R64 | 1 | *Flavobacterium resistens* | NR_044292 | 97 | 672/693 |
| R | A-8R41 | 1 | *Microbacterium flavescens* | JF778699 | 99 | 837/849 |
| R | A-8R53 | 1 | *Microbacterium flavescens* | HQ530520 | 99 | 692/702 |
| R | A-8R78 | 1 | *Microbacterium arthrosphaerae* | KF876895 | 99 | 798/801 |
| R | A-8R17 | 1 | *Pseudomonas putida* | DQ133506 | 99 | 595/597 |
| R | A-8R18 | 1 | *Pseudomonas frederiksbergensis* | KT634069 | 99 | 798/799 |
| R | A-8R45 | 1 | *Pseudomonas frederiksbergensis* | KT634069 | 100 | 798/798 |
| R | A-8R48 | 1 | *Pseudomonas frederiksbergensis* | KT634069 | 99 | 678/687 |
| R | A-8R50B | 2 | *Pseudomonas frederiksbergensis* | KT634069 | 100 | 799/799 |
| R | A-8R51 | 1 | *Pseudomonas frederiksbergensis* | KT634069 | 99 | 643/647 |
| R | A-8R67 | 2 | *Pseudomonas brassicacearum* | KT997466 | 100 | 1000/1000 |
| R | A-8R58 | 1 | *Pseudomonas brassicacearum* | KY203804 | 100 | 844/844 |
| R | A-8R11 | 2 | *Streptomyces drozdowiczii* | KX510091 | 100 | 700/700 |
| R | A-8R36 | 1 | *Streptomyces tuirus* | KX055838 | 99 | 798/800 |
| R | A-8R6 | 3 | *Variovorax paradoxus* | MF101074 | 100 | 798/798 |
| R | A-8R29 | 1 | *Variovorax paradoxus* | KX150839 | 99 | 787/792 |
| R | A-8R33 | 2 | *Variovorax paradoxus* | KX036593 | 100 | 669/669 |
| R | A-8R40 | 1 | *Variovorax paradoxus* | EU169160 | 99 | 737/740 |
| R | A-8R57 | 1 | *Variovorax paradoxus* | FR877674 | 99 | 787/792 |
| R | A-8R61 | 2 | *Variovorax paradoxus* | FR877674 | 99 | 795/797 |
| R | A-8R77 | 1 | *Variovorax paradoxus* | MF101074 | 99 | 849/850 |
| R | A-8R24 | 2 | *Xanthomonas sp. RP-B14* | FM997990 | 99 | 599/600 |
| R | A-8R47 | 1 | *Flavobacterium resistens* | NR_044292 | 98 | 976/996 |
| SSR | A-8S73 | 1 | *Arthrobacter globiformis* | JF496439 | 100 | 850/850 |
| SSR | A-8S42 | 1 | *Arthrobacter crystallopoietes* | KJ542129 | 100 | 800/800 |
| SSR | A-8S61 | 1 | *Arthrobacter crystallopoietes* | KJ542129 | 100 | 800/800 |
| SSR | A-8S21 | 1 | *Bacillus niacini* | KC511548 | 100 | 800/800 |
| SSR | A-8S31 | 2 | *Bacillus simplex* | KY285289 | 99 | 696/699 |
| SSR | A-8S36 | 1 | *Bacillus niacini* | KC511548 | 99 | 797/801 |
| SSR | A-8S41 | 1 | *Brevibacterium frigoritolerans* | MF800956 | 100 | 800/800 |
| SSR | A-8S1 | 1 | *Bacillus firmus* | KC355265 | 98 | 782/802 |
| SSR | A-8S8 | 1 | *Bacillus niacini* | JF496441 | 99 | 749/750 |
| SSR | A-8S24 | 1 | *Bacillus deserti* | NR_117383 | 99 | 793/800 |
| SSR | A-8S16 | 2 | *Brevibacterium frigoritolerans* | MF800956 | 100 | 800/800 |
| SSR | A-8S51 | 1 | *Bacillus deserti* | KT720271 | 99 | 846/849 |
| SSR | A-8S45 | 1 | *Bacillus niacini* | JF682054 | 98 | 719/731 |
| SSR | A-8S48 | 1 | *Bacillus niacini* | HQ284935 | 99 | 799/800 |
| SSR | A-8S55 | 1 | *Brevibacterium frigoritolerans* | KU877654 | 100 | 750/750 |
| SSR | A-8S57 | 1 | *Bacillus niacini* | HF584943 | 99 | 791/800 |
| SSR | A-8S39 | 1 | *Bacillus deserti* | KT720271 | 99 | 794/799 |
| SSR | A-8S59 | 1 | *Brevibacterium frigoritolerans* | MF800956 | 100 | 800/800 |
| SSR | A-8S63 | 1 | *Brevibacterium frigoritolerans* | KU877654 | 98 | 798/800 |
| SSR | A-8S79 | 1 | *Bacillus deserti* | KT720271 | 99 | 696/700 |
| SSR | A-8S65 | 1 | *Bacillus niacini* | HQ284935 | 99 | 796/801 |
| SSR | A-8S67 | 1 | *Brevibacterium frigoritolerans* | MF101183 | 99 | 798/800 |
| SSR | A-8S68 | 1 | *Bacillus simplex* | KY285289 | 98 | 726/742 |
| SSR | A-8S80 | 2 | *Brevibacterium frigoritolerans* | MF062983 | 99 | 799/800 |
| SSR | A-8S23 | 1 | *Bacillus niacini* | HF585023 | 99 | 797/799 |
| SSR | A-8S72 | 1 | *Brevibacterium frigoritolerans* | MF101183 | 99 | 646/647 |
| SSR | A-8S78 | 1 | *Brevibacterium frigoritolerans* | KY127309 | 99 | 697/698 |
| SSR | A-8S27 | 1 | *Chryseobacterium indoltheticum* | KX249602 | 99 | 556/563 |
| SSR | A-8S40 | 1 | *Chryseobacterium kwangjuense* | NR_108175 | 98 | 684/697 |
| SSR | A-8S4 | 1 | *Chryseobacterium ginsenosidimutans* | NR_108691 | 98 | 695/709 |
| SSR | A-8S15 | 1 | *Flavobacterium xueshanense* | KR085801 | 98 | 682/696 |
| SSR | A-8S26 | 1 | *Dyella yeojuensis* | FN796854 | 99 | 686/692 |
| SSR | A-8S14 | 1 | *Lysobacter antibioticus* | KU613064 | 99 | 696/701 |
| SSR | A-8S6 | 2 | *Arthrobacter crystallopoietes* | HM480372 | 98 | 690/705 |
| SSR | A-8S47 | 1 | *Paenibacillus lautus* | JF496308 | 99 | 898/900 |
| SSR | A-8S2 | 1 | *Paenibacillus lautus* | KU955657 | 99 | 848/850 |
| SSR | A-8S11 | 1 | *Pseudoxanthomonas indica* | JQ659735 | 99 | 697/700 |
| SSR | A-8S9 | 1 | *Sphingomonas ginsengisoli* | NR_132664 | 99 | 694/703 |
| SSR | A-8S62 | 1 | *Stenotrophomonas rhizophila* | JN700143 | 99 | 696/700 |
| SSR | A-8S76 | 1 | *Stenotrophomonas rhizophila* | JN700143 | 100 | 750/750 |
| SSR | A-8S3 | 1 | *Streptomyces flavotricini* | EU593749 | 100 | 700/700 |
| SSR | A-8S10 | 2 | *Streptomyces caeruleatus* | KY908443 | 100 | 800/800 |
| SSR | A-8S35 | 1 | *Streptomyces ostreogriseus* | AB184392 | 99 | 800/802 |
| SSR | A-8S43 | 2 | *Streptomyces caeruleatus* | KY908443 | 100 | 850/850 |
| SSR | A-8S56 | 1 | *Streptomyces bobili* | KP338792 | 100 | 850/850 |
| SSR | A-8S44 | 1 | *Streptomyces caeruleatus* | KF876897 | 100 | 800/800 |
| SSR | A-8S34 | 1 | *Bacteroidetes bacterium ONC1* | FN554385 | 98 | 887/905 |
| SSR | A-8S74 | 1 | *Terribacillus saccharophilus* | KC764977 | 100 | 700/700 |
| SSR | A-8S7 | 1 | *Variovorax paradoxus* | EU169160 | 99 | 796/799 |
| SSR | A-8S12 | 2 | *Variovorax paradoxus* | EU169160 | 100 | 748/748 |
| SSR | A-8S25 | 3 | *Variovorax paradoxus* | KU522201 | 99 | 697/699 |
| SSR | A-8S32 | 4 | *Variovorax boronicumulans* | MF796742 | 99 | 688/694 |
| SSR | A-8S60 | 1 | *Variovorax boronicumulans* | MF796710 | 99 | 699/700 |
| SSR | A-8S64 | 1 | *Variovorax paradoxus* | FR877674 | 99 | 794/798 |
| SSR | A-8S13 | 1 | *Acinetobacter lwoffii* | MF988732 | 100 | 782/782 |
| SSR | A-8S18 | 1 | *Arthrobacter globiformis* | MF620067 | 99 | 840/842 |
| SSR | A-8S29 | 1 | *Variovorax boronicumulans* | CP023284 | 99 | 876/877 |
| B | A-8B73 | 1 | *Agromyces iriomotensis* | KY753307 | 99 | 692/698 |
| B | A-8B1 | 1 | *Arthrobacter oxydans* | HF585024 | 99 | 791/802 |
| B | A-8B26 | 4 | *Arthrobacter oxydans* | KR085876 | 99 | 600/606 |
| B | A-8B78 | 2 | *Pseudarthrobacter siccitolerans* | KY218890 | 99 | 697/701 |
| B | A-8B48 | 1 | *Arthrobacter oxydans* | KC128918 | 100 | 798/798 |
| B | A-8B57 | 1 | *Arthrobacter humicola* | KT382249 | 99 | 898/900 |
| B | A-8B64 | 1 | *Arthrobacter oxydans* | KR085876 | 99 | 799/800 |
| B | A-8B65 | 1 | *Pseudarthrobacter siccitolerans* | KY218890 | 99 | 898/901 |
| B | A-8B66 | 1 | *Arthrobacter oxydans* | JF496330 | 99 | 850/851 |
| B | A-8B3 | 2 | *Bacillus wiedmannii* | MF988727 | 99 | 645/650 |
| B | A-8B5 | 2 | *Bacillus thuringiensis* | KY419155 | 100 | 749/749 |
| B | A-8B7 | 1 | *Bacillus cereus* | KY312802 | 99 | 799/800 |
| B | A-8B8 | 1 | *Bacillus massiliosenegalensis* | NR_125590 | 98 | 784/796 |
| B | A-8B22 | 1 | *Brevibacterium frigoritolerans* | KU877654 | 100 | 800/800 |
| B | A-8B28 | 1 | *Bacillus deserti* | KT720271 | 99 | 847/850 |
| B | A-8B29 | 1 | *Bacillus cereus* | KY312801 | 99 | 799/801 |
| B | A-8B20 | 3 | *Bacillus cereus* | KY312801 | 99 | 849/850 |
| B | A-8B35 | 2 | *Bacillus niacini* | HQ284935 | 99 | 699/700 |
| B | A-8B76 | 2 | *Bacillus niacini* | KC511548 | 99 | 647/650 |
| B | A-8B80 | 4 | *Bacillus niacini* | KC315764 | 100 | 950/950 |
| B | A-8B51 | 1 | *Bacillus niacini* | HQ284935 | 99 | 699/700 |
| B | A-8B56 | 2 | *Bacillus niacini* | KT720170 | 99 | 795/796 |
| B | A-8B62 | 1 | *Bacillus badius* | KY950621 | 97 | 635/654 |
| B | A-8B72 | 1 | *Bacillus niacini* | HQ284935 | 99 | 647/650 |
| B | A-8B2 | 3 | *Bacillus cereus* | KM977832 | 99 | 798/800 |
| B | A-8B36B | 1 | *Bacillus cereus* | KM977832 | 99 | 509/513 |
| B | A-8B70 | 1 | *Massilia niastensis* | JF496359 | 99 | 816/824 |
| B | A-8B46 | 2 | *Microbacterium hydrocarbonoxydans* | EU714352 | 99 | 799/801 |
| B | A-8B11 | 1 | *Arthrobacter humicola* | KT382249 | 99 | 716/725 |
| B | A-8B67 | 1 | *Staphylococcus warneri* | MG027625 | 100 | 845/845 |
| B | A-8B6 | 2 | *Streptomyces fragilis* | EU841657 | 99 | 848/850 |
| B | A-8B13 | 1 | *Streptomyces gobitricini* | KU884395 | 99 | 798/800 |
| B | A-8B16 | 1 | *Streptomyces fragilis* | KM186631 | 99 | 848/850 |
| B | A-8B18 | 1 | *Streptomyces venezuelae* | KY007173 | 99 | 899/900 |
| B | A-8B24 | 1 | *Streptomyces fragilis* | KX352763 | 99 | 898/900 |
| B | A-8B25 | 1 | *Streptomyces bottropensis* | KY753283 | 99 | 702/706 |
| B | A-8B31 | 3 | *Streptomyces fragilis* | KM186631 | 99 | 899/901 |
| B | A-8B34 | 2 | *Streptomyces tumescens* | KU647226 | 100 | 851/851 |
| B | A-8B47 | 1 | *Streptomyces nojiriensis* | KX380878 | 100 | 900/900 |
| B | A-8B54 | 3 | *Streptomyces fragilis* | KM186631 | 99 | 899/901 |
| B | A-8B43 | 1 | *Streptomyces fragilis* | KM186631 | 99 | 894/898 |
| B | A-8B41 | 1 | *Streptomyces flavovariabilis* | FJ792572 | 99 | 845/852 |
| B | A-8B79 | 2 | *Streptomyces flavovariabilis* | FJ792572 | 99 | 801/808 |
| B | A-8B49 | 1 | *Streptomyces luteoverticillatus* | KU310977 | 99 | 896/900 |
| B | A-8B50 | 1 | *Streptomyces hawaiiensis* | EU624140 | 100 | 900/900 |
| B | A-8B60 | 1 | *Streptomyces tumescens* | KU647226 | 100 | 902/902 |
| B | A-8B69 | 1 | *Streptomyces cinnamocastaneus* | AB184588 | 99 | 698/699 |
| B | A-8B71 | 1 | *Streptomyces ciscaucasicus* | KY753363 | 99 | 662/663 |
| B | A-8B74 | 1 | *Streptomyces scabiei* | KP718596 | 99 | 646/650 |
| B | A-8B12 | 1 | *Variovorax paradoxus* | MF101074 | 99 | 746/747 |
| B | A-8B63 | 1 | *Streptomyces atroolivaceus* | KY744653 | 100 | 605/605 |
| Re | A-8SB13 | 1 | *Streptomyces flavovariabilis* | FJ792572 | 98 | 903/926 |
| Re | A-8SB20 | 1 | *Streptomyces tumescens* | KU647226 | 98 | 786/805 |
| Re | A-8SB18 | 1 | *Arthrobacter globiformis* | KY859819 | 99 | 897/902 |
| Re | A-8SB41 | 1 | *Arthrobacter crystallopoietes* | KY753214 | 98 | 935/950 |
| Re | A-8SB2 | 1 | *Bacillus megaterium* | KT764108 | 98 | 877/893 |
| Re | A-8SB3 | 3 | *Bacillus megaterium* | FJ174652 | 99 | 960/961 |
| Re | A-8SB5 | 7 | *Bacillus simplex* | KF478196 | 99 | 859/865 |
| Re | A-8SB9 | 1 | *Brevibacterium frigoritolerans* | MF620091 | 100 | 596/596 |
| Re | A-8SB12 | 2 | *Bacillus niacini* | KT720170 | 99 | 891/893 |
| Re | A-8SB14 | 1 | *Bacillus simplex* | KY646079 | 99 | 893/899 |
| Re | A-8SB25 | 1 | *Bacillus megaterium* | KC764961 | 100 | 989/989 |
| Re | A-8SB29 | 2 | *Bacillus cereus* | KP411923 | 100 | 899/899 |
| Re | A-8SB43 | 1 | *Bacillus simplex* | JF496503 | 99 | 753/764 |
| Re | A-8SB22 | 1 | *Variovorax paradoxus* | FR877674 | 100 | 848/848 |
| Re | A-8SB4 | 3 | *Chryseobacterium ginsenosidimutans* | KU924004 | 97 | 831/858 |
| Re | A-8SB73 | 1 | *Chryseobacterium ginsenosidimutans* | KU924004 | 97 | 823/846 |
| Re | A-8SB6 | 1 | *Flavobacterium xueshanense* | KR085801 | 98 | 842/861 |
| Re | A-8SB7 | 1 | *Flavobacterium spartansii* | NR_133748 | 98 | 782/799 |
| Re | A-8SB8 | 1 | *Flavobacterium aquidurense* | NR_042470 | 98 | 932/949 |
| Re | A-8SB15 | 1 | *Flavobacterium gyeonganense* | NR_134035 | 98 | 857/874 |
| Re | A-8SB16 | 1 | *Flavobacterium pectinovorum* | NR_114994 | 97 | 882/912 |
| Re | A-8SB19 | 1 | *Flavobacterium xueshanense* | KR085801 | 98 | 975/995 |
| Re | A-8SB21 | 1 | *Flavobacterium xueshanense* | KR085801 | 98 | 877/897 |
| Re | A-8SB42 | 1 | *Flavobacterium pectinovorum* | NR_114994 | 98 | 983/1003 |
| Re | A-8SB57 | 1 | *Flavobacterium chungangense* | NR_044581 | 98 | 872/890 |
| Re | A-8SB59 | 2 | *Flavobacterium chungangense* | NR_044581 | 98 | 872/893 |
| Re | A-8SB77 | 1 | *Flavobacterium resistens* | NR_044292 | 98 | 780/799 |
| Re | A-8SB70 | 1 | *Flavobacterium chungangense* | NR_044581 | 97 | 803/824 |
| Re | A-8SB69 | 1 | *Flavobacterium aquidurense* | NR_042470 | 98 | 781/797 |
| Re | A-8SB33 | 1 | *Flavobacterium procerum* | NR_136820 | 97 | 772/801 |
| Re | A-8SB45 | 1 | *Flavobacterium arsenitoxidans* | LN995689 | 97 | 871/900 |
| Re | A-8SB49 | 1 | *Lysobacter antibioticus* | CP011129 | 99 | 995/999 |
| Re | A-8SB26 | 4 | *Lysobacter antibioticus* | CP011129 | 99 | 797/798 |
| Re | A-8SB48 | 1 | *Massilia niastensis* | JF496359 | 98 | 987/1003 |
| Re | A-8SB53 | 1 | *Microbacterium oxydans* | KR085856 | 99 | 996/1000 |
| Re | A-8SB75 | 1 | *Microbacterium invictum* | NR_042708 | 98 | 793/806 |
| Re | A-8SB54 | 1 | *Microbacterium flavescens* | EU714363 | 99 | 693/694 |
| Re | A-8SB34 | 1 | *Paracoccus yeei* | CP020442 | 100 | 849/849 |
| Re | A-8SB40 | 1 | *Pseudomonas helmanticensis* | KY457748 | 99 | 898/899 |
| Re | A-8SB66 | 1 | *Pseudomonas alcaligenes* | JF915337 | 99 | 939/944 |
| Re | A-8SB1 | 2 | *Streptomyces spororaveus* | KX035075 | 99 | 949/959 |
| Re | A-8SB23 | 1 | *Streptomyces nojiriensis* | KX380878 | 99 | 900/901 |
| Re | A-8SB24 | 1 | *Streptomyces ambofaciens* | KT363055 | 100 | 916/916 |
| Re | A-8SB32 | 1 | *Streptomyces pluricolorescens* | KU324442 | 99 | 906/912 |
| Re | A-8SB31 | 2 | *Streptomyces flavovariabilis* | FJ792572 | 99 | 891/899 |
| Re | A-8SB35 | 1 | *Streptomyces netropsis* | KP339302 | 99 | 749/760 |
| Re | A-8SB36 | 1 | *Streptomyces ambofaciens* | EU841656 | 99 | 912/919 |
| Re | A-8SB51 | 1 | *Streptomyces gardneri* | JN999909 | 98 | 1075/1094 |
| Re | A-8SB52 | 1 | *Streptomyces venezuelae* | KY007173 | 97 | 647/664 |
| Re | A-8SB58 | 1 | *Streptomyces rectiviolaceus* | KJ081286 | 99 | 744/749 |
| Re | A-8SB63 | 1 | *Streptomyces ambofaciens* | EU593561 | 100 | 849/849 |
| Re | A-8SB44 | 1 | *Streptomyces virginiae* | KU317910 | 100 | 949/949 |
| Re | A-8SB55 | 1 | *Variovorax paradoxus* | KX150846 | 97 | 952/980 |
| Re | A-8SB72 | 1 | *Variovorax paradoxus* | FR877674 | 99 | 882/893 |
| Re | A-8SB80 | 1 | *Variovorax paradoxus* | KX036607 | 100 | 749/749 |
| Re | A-8SB10 | 1 | *Xanthomonas translucens* | GU902285 | 99 | 796/799 |
| Re | A-8SB11 | 1 | *Xanthomonas translucens* | GU902285 | 99 | 896/899 |
| Re | A-8SB38 | 1 | *Xanthomonas translucens* | GU902285 | 99 | 750/759 |
| Re | A-8SB71 | 1 | *Flavobacterium ginsengisoli* | KY077156 | 97 | 834/863 |

**Supplementary Table 3. Plant growth promotion traits of the cultivable bacteria isolated from the root apparatus of *A. spinosa* (E, R, SSR), bulk soil (B) and residuesphere (Re).** The table includes the results of the screening performed to characterize the *in vitro* direct and indirect PGP potential of the bacterial strains. Sid: siderophore production; PO_4_: phosphate solubilization activity; NH_3_: ammonia production; EPS: exopolysaccharides. For each activity, 0/1 indicate the absence or presence in the isolates.

| **Strain** | **PO_4_ sol.** | | **Sid** | | **NH_3_** | | **Protease** | | **Esterase** | | **EPS** | |  |
| --- | --- | --- | --- | --- | --- | --- | --- | --- | --- | --- | --- | --- | --- |
| A-8E1 | | 1 | | 0 | | 1 | | 1 | | 0 | | 0 | |
| A-8E16 | | 0 | | 0 | | 1 | | 0 | | 0 | | 1 | |
| A-8E2 | | 0 | | 0 | | 1 | | 0 | | 0 | | 0 | |
| A-8E26 | | 0 | | 0 | | 1 | | 1 | | 0 | | 0 | |
| A-8E29 | | 0 | | 0 | | 1 | | 1 | | 0 | | 0 | |
| A-8E31 | | 1 | | 0 | | 1 | | 0 | | 0 | | 0 | |
| A-8E54 | | 0 | | 0 | | 1 | | 1 | | 0 | | 0 | |
| A-8E57 | | 0 | | 0 | | 1 | | 0 | | 0 | | 0 | |
| A-8E62 | | 0 | | 0 | | 1 | | 1 | | 0 | | 0 | |
| A-8E73 | | 0 | | 0 | | 1 | | 0 | | 1 | | 0 | |
| A-8E74 | | 0 | | 0 | | 0 | | 0 | | 0 | | 0 | |
| A-8R01 | | 0 | | 0 | | 1 | | 1 | | 0 | | 0 | |
| A-8R02 | | 0 | | 0 | | 1 | | 1 | | 0 | | 0 | |
| A-8R04 | | 0 | | 0 | | 1 | | 0 | | 1 | | 0 | |
| A-8R05 | | 0 | | 0 | | 0 | | 1 | | 0 | | 0 | |
| A-8R06 | | 0 | | 0 | | 1 | | 0 | | 0 | | 0 | |
| A-8R07 | | 0 | | 0 | | 1 | | 1 | | 0 | | 0 | |
| A-8R09 | | 0 | | 0 | | 1 | | 1 | | 0 | | 0 | |
| A-8R10 | | 0 | | 0 | | 1 | | 1 | | 0 | | 0 | |
| A-8R11 | | 0 | | 0 | | 0 | | 1 | | 0 | | 0 | |
| A-8R12 | | 0 | | 0 | | 1 | | 0 | | 0 | | 0 | |
| A-8R13 | | 0 | | 0 | | 0 | | 0 | | 0 | | 0 | |
| A-8R14 | | 0 | | 0 | | 1 | | 1 | | 0 | | 0 | |
| A-8R17 | | 0 | | 1 | | 1 | | 0 | | 0 | | 0 | |
| A-8R18 | | 1 | | 1 | | 1 | | 0 | | 0 | | 0 | |
| A-8R20 | | 0 | | 0 | | 1 | | 0 | | 0 | | 0 | |
| A-8R21 | | 0 | | 0 | | 1 | | 1 | | 0 | | 0 | |
| A-8R22 | | 0 | | 0 | | 1 | | 1 | | 0 | | 0 | |
| A-8R23 | | 0 | | 0 | | 1 | | 1 | | 0 | | 0 | |
| A-8R24 | | 0 | | 0 | | 1 | | 1 | | 1 | | 0 | |
| A-8R28 | | 0 | | 0 | | 1 | | 1 | | 0 | | 0 | |
| A-8R29 | | 0 | | 0 | | 1 | | 0 | | 0 | | 0 | |
| A-8R30 | | 0 | | 0 | | 1 | | 0 | | 0 | | 0 | |
| A-8R32 | | 0 | | 0 | | 1 | | 1 | | 0 | | 0 | |
| A-8R33 | | 0 | | 0 | | 1 | | 0 | | 1 | | 0 | |
| A-8R36 | | 0 | | 0 | | 0 | | 0 | | 0 | | 0 | |
| A-8R37 | | 0 | | 0 | | 0 | | 1 | | 0 | | 0 | |
| A-8R38 | | 0 | | 0 | | 1 | | 1 | | 1 | | 0 | |
| A-8R40 | | 0 | | 0 | | 1 | | 0 | | 0 | | 0 | |
| A-8R41 | | 0 | | 0 | | 1 | | 0 | | 1 | | 0 | |
| A-8R43A | | 0 | | 0 | | 1 | | 0 | | 0 | | 0 | |
| A-8R44 | | 0 | | 0 | | 1 | | 1 | | 0 | | 0 | |
| A-8R45 | | 1 | | 1 | | 1 | | 0 | | 0 | | 0 | |
| A-8R47 | | 1 | | 1 | | 1 | | 0 | | 0 | | 0 | |
| A-8R48 | | 1 | | 1 | | 1 | | 0 | | 0 | | 0 | |
| A-8R50B | | 0 | | 0 | | 1 | | 0 | | 0 | | 0 | |
| A-8R51 | | 0 | | 0 | | 1 | | 1 | | 0 | | 0 | |
| A-8R52 | | 0 | | 0 | | 1 | | 0 | | 0 | | 0 | |
| A-8R53 | | 0 | | 1 | | 1 | | 0 | | 0 | | 0 | |
| A-8R54 | | 0 | | 0 | | 1 | | 1 | | 0 | | 0 | |
| A-8R57 | | 0 | | 0 | | 1 | | 0 | | 0 | | 0 | |
| A-8R58 | | 1 | | 1 | | 1 | | 0 | | 1 | | 0 | |
| A-8R60 | | 0 | | 0 | | 0 | | 1 | | 0 | | 0 | |
| A-8R61 | | 0 | | 0 | | 1 | | 0 | | 0 | | 0 | |
| A-8R64 | | 0 | | 0 | | 0 | | 0 | | 0 | | 0 | |
| A-8R65 | | 0 | | 0 | | 0 | | 0 | | 1 | | 0 | |
| A-8R67 | | 0 | | 1 | | 0 | | 1 | | 0 | | 0 | |
| A-8R69 | | 0 | | 0 | | 1 | | 0 | | 0 | | 0 | |
| A-8R77 | | 0 | | 0 | | 0 | | 0 | | 0 | | 0 | |
| A-8R78 | | 0 | | 0 | | 1 | | 0 | | 0 | | 0 | |
| A-8S01 | | 0 | | 0 | | 1 | | 0 | | 0 | | 0 | |
| A-8S02 | | 0 | | 0 | | 0 | | 0 | | 0 | | 0 | |
| A-8S03 | | 1 | | 1 | | 1 | | 0 | | 0 | | 0 | |
| A-8S04 | | 0 | | 1 | | 1 | | 1 | | 0 | | 0 | |
| A-8S06 | | 1 | | 1 | | 1 | | 0 | | 0 | | 0 | |
| A-8S07 | | 1 | | 0 | | 1 | | 1 | | 0 | | 0 | |
| A-8S08 | | 0 | | 1 | | 1 | | 1 | | 0 | | 0 | |
| A-8S09 | | 0 | | 0 | | 1 | | 0 | | 0 | | 0 | |
| A-8S10 | | 1 | | 1 | | 1 | | 1 | | 0 | | 0 | |
| A-8S11 | | 0 | | 1 | | 1 | | 1 | | 0 | | 0 | |
| A-8S12 | | 0 | | 1 | | 1 | | 0 | | 1 | | 0 | |
| A-8S13 | | 1 | | 1 | | 1 | | 0 | | 0 | | 0 | |
| A-8S14 | | 0 | | 1 | | 1 | | 0 | | 0 | | 0 | |
| A-8S15 | | 0 | | 1 | | 1 | | 1 | | 0 | | 0 | |
| A-8S16 | | 0 | | 0 | | 1 | | 1 | | 0 | | 0 | |
| A-8S18 | | 1 | | 0 | | 1 | | 1 | | 0 | | 0 | |
| A-8S21 | | 1 | | 1 | | 1 | | 0 | | 0 | | 0 | |
| A-8S23 | | 0 | | 1 | | 1 | | 0 | | 0 | | 0 | |
| A-8S24 | | 0 | | 1 | | 0 | | 0 | | 1 | | 0 | |
| A-8S25 | | 1 | | 0 | | 1 | | 0 | | 1 | | 0 | |
| A-8S26 | | 0 | | 0 | | 1 | | 1 | | 0 | | 0 | |
| A-8S27 | | 0 | | 1 | | 1 | | 1 | | 0 | | 0 | |
| A-8S29 | | 1 | | 0 | | 1 | | 1 | | 0 | | 0 | |
| A-8S31 | | 0 | | 0 | | 1 | | 0 | | 0 | | 0 | |
| A-8S32 | | 1 | | 0 | | 1 | | 0 | | 0 | | 0 | |
| A-8S34 | | 0 | | 1 | | 1 | | 0 | | 1 | | 0 | |
| A-8S35 | | 0 | | 0 | | 1 | | 0 | | 0 | | 0 | |
| A-8S36 | | 0 | | 1 | | 1 | | 0 | | 0 | | 0 | |
| A-8S39 | | 0 | | 0 | | 1 | | 0 | | 0 | | 0 | |
| A-8S40 | | 0 | | 1 | | 1 | | 1 | | 0 | | 0 | |
| A-8S41 | | 0 | | 1 | | 1 | | 1 | | 0 | | 0 | |
| A-8S42 | | 1 | | 0 | | 1 | | 0 | | 0 | | 0 | |
| A-8S43 | | 0 | | 0 | | 1 | | 0 | | 0 | | 0 | |
| A-8S44 | | 0 | | 1 | | 0 | | 1 | | 0 | | 0 | |
| A-8S45 | | 0 | | 0 | | 1 | | 1 | | 0 | | 0 | |
| A-8S47 | | 0 | | 0 | | 0 | | 0 | | 0 | | 0 | |
| A-8S48 | | 0 | | 0 | | 1 | | 0 | | 0 | | 0 | |
| A-8S51 | | 0 | | 0 | | 1 | | 0 | | 0 | | 0 | |
| A-8S55 | | 0 | | 0 | | 1 | | 1 | | 0 | | 0 | |
| A-8S56 | | 0 | | 1 | | 1 | | 0 | | 0 | | 0 | |
| A-8S57 | | 0 | | 0 | | 1 | | 1 | | 0 | | 0 | |
| A-8S59 | | 1 | | 0 | | 1 | | 0 | | 0 | | 0 | |
| A-8S60 | | 1 | | 1 | | 1 | | 0 | | 0 | | 0 | |
| A-8S61 | | 0 | | 0 | | 1 | | 1 | | 0 | | 0 | |
| A-8S62 | | 0 | | 0 | | 1 | | 1 | | 0 | | 0 | |
| A-8S63 | | 0 | | 0 | | 1 | | 1 | | 0 | | 0 | |
| A-8S64 | | 1 | | 0 | | 1 | | 1 | | 0 | | 0 | |
| A-8S65 | | 0 | | 0 | | 1 | | 0 | | 0 | | 0 | |
| A-8S67 | | 0 | | 0 | | 1 | | 1 | | 0 | | 0 | |
| A-8S68 | | 0 | | 1 | | 1 | | 1 | | 0 | | 0 | |
| A-8S72 | | 0 | | 0 | | 1 | | 1 | | 0 | | 0 | |
| A-8S73 | | 0 | | 1 | | 1 | | 0 | | 0 | | 0 | |
| A-8S74 | | 0 | | 0 | | 1 | | 0 | | 0 | | 0 | |
| A-8S76 | | 0 | | 0 | | 1 | | 1 | | 0 | | 0 | |
| A-8S78 | | 1 | | 0 | | 1 | | 1 | | 0 | | 0 | |
| A-8S79 | | 0 | | 0 | | 1 | | 0 | | 0 | | 0 | |
| A-8S80 | | 0 | | 1 | | 1 | | 1 | | 0 | | 0 | |
| A-8B01 | | 0 | | 0 | | 1 | | 1 | | 0 | | 0 | |
| A-8B02 | | 0 | | 0 | | 1 | | 1 | | 0 | | 0 | |
| A-8B03 | | 0 | | 1 | | 1 | | 0 | | 0 | | 0 | |
| A-8B05 | | 0 | | 0 | | 1 | | 1 | | 0 | | 0 | |
| A-8B06 | | 0 | | 0 | | 1 | | 0 | | 0 | | 0 | |
| A-8B07 | | 0 | | 0 | | 1 | | 0 | | 0 | | 0 | |
| A-8B08 | | 0 | | 0 | | 1 | | 1 | | 1 | | 0 | |
| A-8B11 | | 0 | | 1 | | 1 | | 0 | | 0 | | 0 | |
| A-8B12 | | 0 | | 0 | | 0 | | 0 | | 0 | | 0 | |
| A-8B13 | | 0 | | 0 | | 1 | | 0 | | 0 | | 0 | |
| A-8B16 | | 0 | | 0 | | 1 | | 0 | | 0 | | 0 | |
| A-8B18 | | 0 | | 0 | | 1 | | 0 | | 0 | | 0 | |
| A-8B20 | | 0 | | 1 | | 1 | | 0 | | 0 | | 0 | |
| A-8B22 | | 0 | | 0 | | 1 | | 0 | | 0 | | 0 | |
| A-8B24 | | 0 | | 0 | | 1 | | 1 | | 0 | | 0 | |
| A-8B25 | | 0 | | 0 | | 1 | | 0 | | 0 | | 0 | |
| A-8B26 | | 0 | | 0 | | 1 | | 1 | | 0 | | 0 | |
| A-8B28 | | 0 | | 1 | | 1 | | 1 | | 0 | | 0 | |
| A-8B29 | | 0 | | 0 | | 1 | | 1 | | 0 | | 0 | |
| A-8B31 | | 0 | | 0 | | 0 | | 0 | | 0 | | 0 | |
| A-8B34 | | 0 | | 0 | | 1 | | 0 | | 0 | | 0 | |
| A-8B36B | | 0 | | 0 | | 1 | | 1 | | 0 | | 0 | |
| A-8B41 | | 0 | | 1 | | 1 | | 1 | | 0 | | 0 | |
| A-8B43 | | 0 | | 0 | | 1 | | 1 | | 0 | | 0 | |
| A-8B46 | | 0 | | 0 | | 1 | | 1 | | 0 | | 0 | |
| A-8B47 | | 0 | | 1 | | 1 | | 0 | | 1 | | 0 | |
| A-8B48 | | 0 | | 0 | | 1 | | 0 | | 0 | | 0 | |
| A-8B49 | | 0 | | 0 | | 1 | | 1 | | 0 | | 0 | |
| A-8B50 | | 0 | | 0 | | 0 | | 0 | | 0 | | 0 | |
| A-8B51 | | 0 | | 0 | | 1 | | 1 | | 0 | | 0 | |
| A-8B54 | | 0 | | 1 | | 1 | | 1 | | 0 | | 0 | |
| A-8B56 | | 0 | | 0 | | 1 | | 0 | | 0 | | 0 | |
| A-8B57 | | 0 | | 1 | | 1 | | 0 | | 0 | | 0 | |
| A-8B60 | | 0 | | 0 | | 1 | | 1 | | 0 | | 0 | |
| A-8B62 | | 0 | | 0 | | 1 | | 0 | | 0 | | 0 | |
| A-8B63 | | 0 | | 0 | | 1 | | 1 | | 0 | | 0 | |
| A-8B64 | | 0 | | 1 | | 1 | | 1 | | 0 | | 0 | |
| A-8B65 | | 0 | | 0 | | 1 | | 1 | | 0 | | 0 | |
| A-8B66 | | 0 | | 0 | | 1 | | 1 | | 0 | | 0 | |
| A-8B67 | | 0 | | 1 | | 1 | | 0 | | 0 | | 0 | |
| A-8B69 | | 0 | | 1 | | 1 | | 0 | | 1 | | 0 | |
| A-8B70 | | 0 | | 1 | | 1 | | 0 | | 1 | | 0 | |
| A-8B71 | | 0 | | 1 | | 1 | | 0 | | 1 | | 0 | |
| A-8B72 | | 0 | | 1 | | 1 | | 0 | | 1 | | 0 | |
| A-8B73 | | 0 | | 0 | | 1 | | 0 | | 0 | | 0 | |
| A-8B74 | | 0 | | 0 | | 1 | | 1 | | 0 | | 0 | |
| A-8B76 | | 0 | | 0 | | 1 | | 1 | | 0 | | 0 | |
| A-8B78 | | 0 | | 0 | | 1 | | 1 | | 0 | | 0 | |
| A-8B80 | | 0 | | 0 | | 0 | | 0 | | 0 | | 0 | |
| A-8SB01 | | 1 | | 0 | | 1 | | 0 | | 1 | | 0 | |
| A-8SB02 | | 1 | | 0 | | 0 | | 1 | | 1 | | 0 | |
| A-8SB03 | | 1 | | 0 | | 1 | | 1 | | 1 | | 0 | |
| A-8SB04 | | 0 | | 0 | | 1 | | 1 | | 0 | | 0 | |
| A-8SB05 | | 0 | | 0 | | 1 | | 0 | | 1 | | 0 | |
| A-8SB06 | | 0 | | 0 | | 1 | | 1 | | 0 | | 0 | |
| A-8SB07 | | 0 | | 0 | | 1 | | 1 | | 0 | | 0 | |
| A-8SB08 | | 0 | | 0 | | 1 | | 1 | | 0 | | 0 | |
| A-8SB09 | | 0 | | 0 | | 1 | | 1 | | 1 | | 0 | |
| A-8SB10 | | 0 | | 0 | | 1 | | 1 | | 1 | | 0 | |
| A-8SB11 | | 0 | | 0 | | 1 | | 1 | | 0 | | 0 | |
| A-8SB12 | | 0 | | 0 | | 1 | | 1 | | 0 | | 0 | |
| A-8SB14 | | 1 | | 1 | | 1 | | 1 | | 0 | | 0 | |
| A-8SB15 | | 0 | | 0 | | 1 | | 1 | | 0 | | 0 | |
| A-8SB16 | | 0 | | 0 | | 1 | | 1 | | 0 | | 0 | |
| A-8SB18 | | 0 | | 0 | | 1 | | 0 | | 0 | | 0 | |
| A-8SB19 | | 0 | | 0 | | 1 | | 1 | | 0 | | 0 | |
| A-8SB21 | | 0 | | 0 | | 1 | | 1 | | 0 | | 0 | |
| A-8SB22 | | 0 | | 1 | | 1 | | 1 | | 0 | | 0 | |
| A-8SB23 | | 0 | | 1 | | 1 | | 1 | | 0 | | 0 | |
| A-8SB24 | | 0 | | 1 | | 1 | | 0 | | 1 | | 0 | |
| A-8SB25 | | 0 | | 0 | | 1 | | 1 | | 1 | | 0 | |
| A-8SB29 | | 0 | | 1 | | 1 | | 1 | | 1 | | 0 | |
| A-8SB32 | | 0 | | 1 | | 1 | | 1 | | 1 | | 0 | |
| A-8SB33 | | 0 | | 0 | | 1 | | 0 | | 1 | | 0 | |
| A-8SB34 | | 0 | | 0 | | 1 | | 0 | | 1 | | 0 | |
| A-8SB35 | | 0 | | 1 | | 1 | | 0 | | 1 | | 0 | |
| A-8SB36 | | 0 | | 0 | | 1 | | 0 | | 1 | | 0 | |
| A-8SB38 | | 0 | | 0 | | 1 | | 1 | | 1 | | 0 | |
| A-8SB40 | | 0 | | 0 | | 1 | | 0 | | 0 | | 0 | |
| A-8SB41 | | 0 | | 1 | | 1 | | 0 | | 1 | | 0 | |
| A-8SB42 | | 0 | | 0 | | 1 | | 0 | | 0 | | 0 | |
| A-8SB43 | | 0 | | 0 | | 1 | | 0 | | 1 | | 0 | |
| A-8SB44 | | 0 | | 1 | | 1 | | 0 | | 0 | | 0 | |
| A-8SB45 | | 0 | | 0 | | 1 | | 1 | | 0 | | 0 | |
| A-8SB48 | | 0 | | 0 | | 1 | | 1 | | 0 | | 0 | |
| A-8SB49 | | 0 | | 1 | | 1 | | 1 | | 1 | | 0 | |
| A-8SB51 | | 0 | | 0 | | 1 | | 0 | | 0 | | 0 | |
| A-8SB52 | | 0 | | 0 | | 1 | | 1 | | 0 | | 0 | |
| A-8SB53 | | 0 | | 1 | | 1 | | 1 | | 1 | | 0 | |
| A-8SB55 | | 1 | | 0 | | 1 | | 0 | | 0 | | 0 | |
| A-8SB57 | | 0 | | 0 | | 1 | | 1 | | 0 | | 0 | |
| A-8SB58 | | 0 | | 0 | | 1 | | 0 | | 0 | | 0 | |
| A-8SB59 | | 0 | | 0 | | 1 | | 1 | | 0 | | 0 | |
| A-8SB63 | | 0 | | 1 | | 1 | | 1 | | 0 | | 0 | |
| A-8SB69 | | 0 | | 0 | | 1 | | 1 | | 0 | | 0 | |
| A-8SB70 | | 0 | | 1 | | 1 | | 0 | | 0 | | 0 | |
| A-8SB71 | | 0 | | 0 | | 1 | | 1 | | 0 | | 0 | |
| A-8SB72 | | 0 | | 1 | | 1 | | 1 | | 1 | | 0 | |
| A-8SB73 | | 0 | | 0 | | 1 | | 1 | | 1 | | 0 | |
| A-8SB75 | | 0 | | 0 | | 1 | | 0 | | 0 | | 0 | |
| A-8SB77 | | 0 | | 0 | | 1 | | 0 | | 0 | | 0 | |
| A-8SB80 | | 0 | | 1 | | 1 | | 0 | | 0 | | 0 | |
